# Supplementary material for: Enhancing the performance of Magnets photosensors
Source: Nat Commun. 2026 Mar 18;17:4138. doi: 10.1038/s41467-026-70695-7 (PMC13149671; doi:10.1038/s41467-026-70695-7)
Supplement: Supplementary file 1 — Supplementary Information [file 41467_2026_70695_MOESM1_ESM.pdf]

Supplementary Information

for

Enhancing the performance of Magnets photosensors

by

Armin Baumschlager, Yanik Weber, David Cánovas, Sara Dionisi, Mustafa Khammash

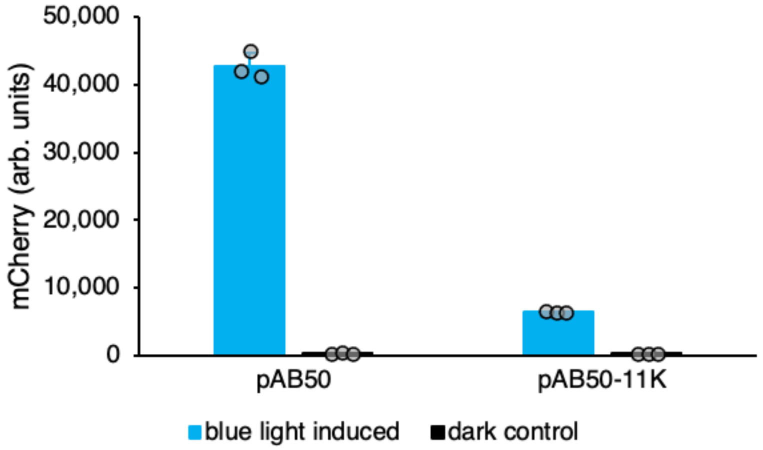

Supplementary Figure 1: Expression level of pAB50 and pAB50-11k with reduced RBS strength using wild-type (WT) Opto-T7RNAP*(563) and saturating light-induction (3.85 W/m^2). Diagram shows mean mCherry expression values and standard deviation (mean values +/- SD) of three (n=3) biological replicates measured after 5h incubation time measured through flow cytometry.

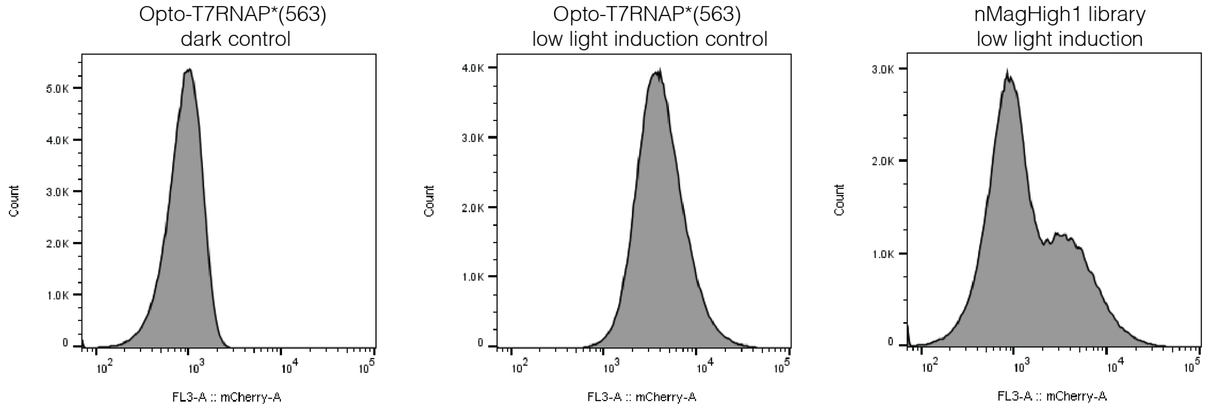

Supplementary Figure 2: mCherry expression level of AB363[1] in the dark (left) and induced with low intensity light (middle) in comparison to the nMagHigh1 library induced with non-saturating (0.96 W m^{-2}) 465-nm light blue light (right). Histograms show mCherry fluorescence obtained during FACS as described in the Methods section.

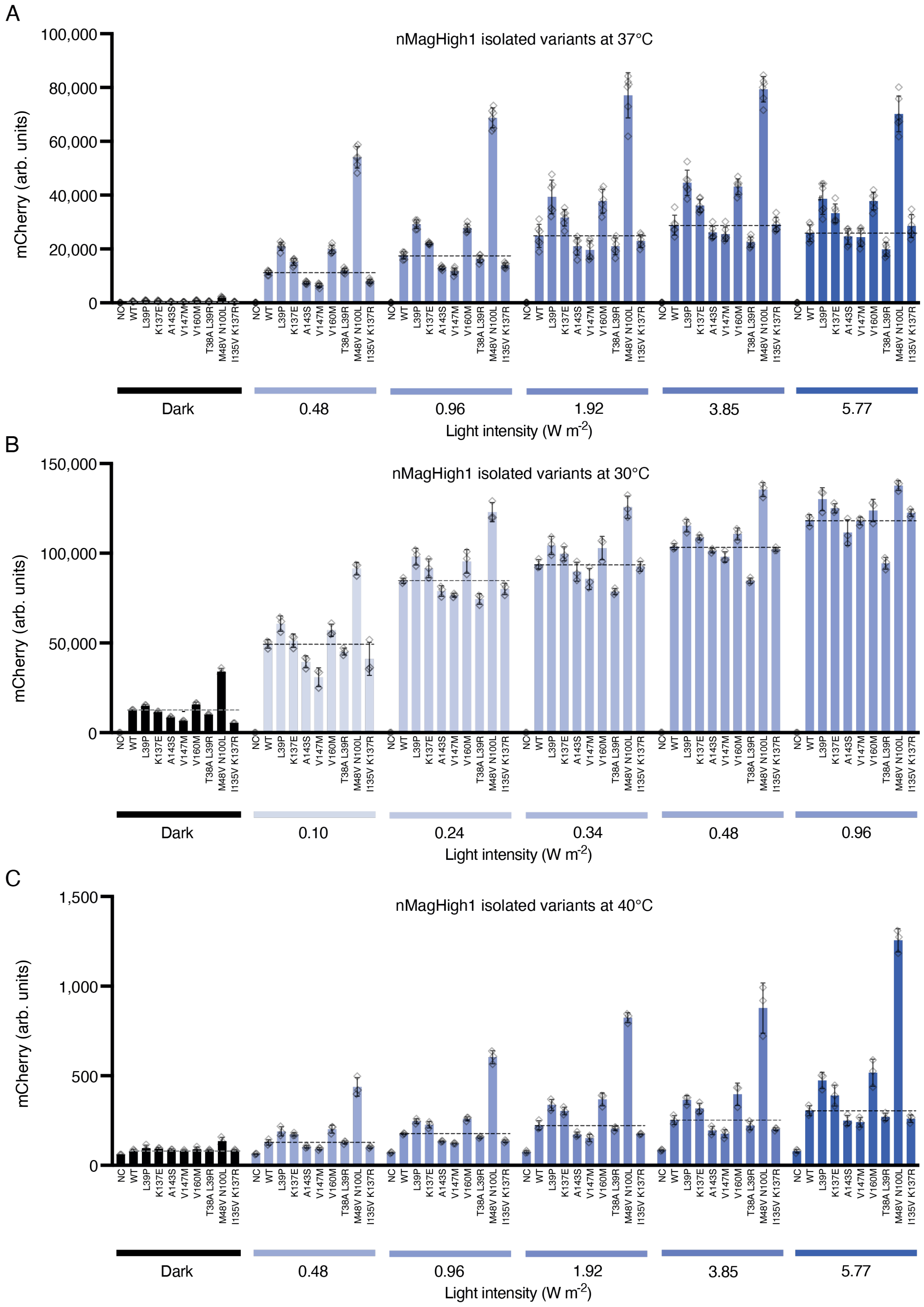

Supplementary Figure 3: Characterization of identified mutations in nMagHigh1 incubated at 37°C (A), 30°C (B) and 40°C (C) through flow cytometry in comparison to the wild-type Opto-T7RNAP*(563) regulator and a negative control (NC) containing the mCherry expression plasmid and a second empty plasmid that does not contain the optogenetic regulator. mCherry expression values were acquired after 5h incubation at the indicated light intensity and temperature. Shown are the mean fluorescence values and standard deviation (mean values +/- SD) as well as individual data points of six (n=6) biological replicates for (A) and three (n=3) biological replicates for (B) and (C).

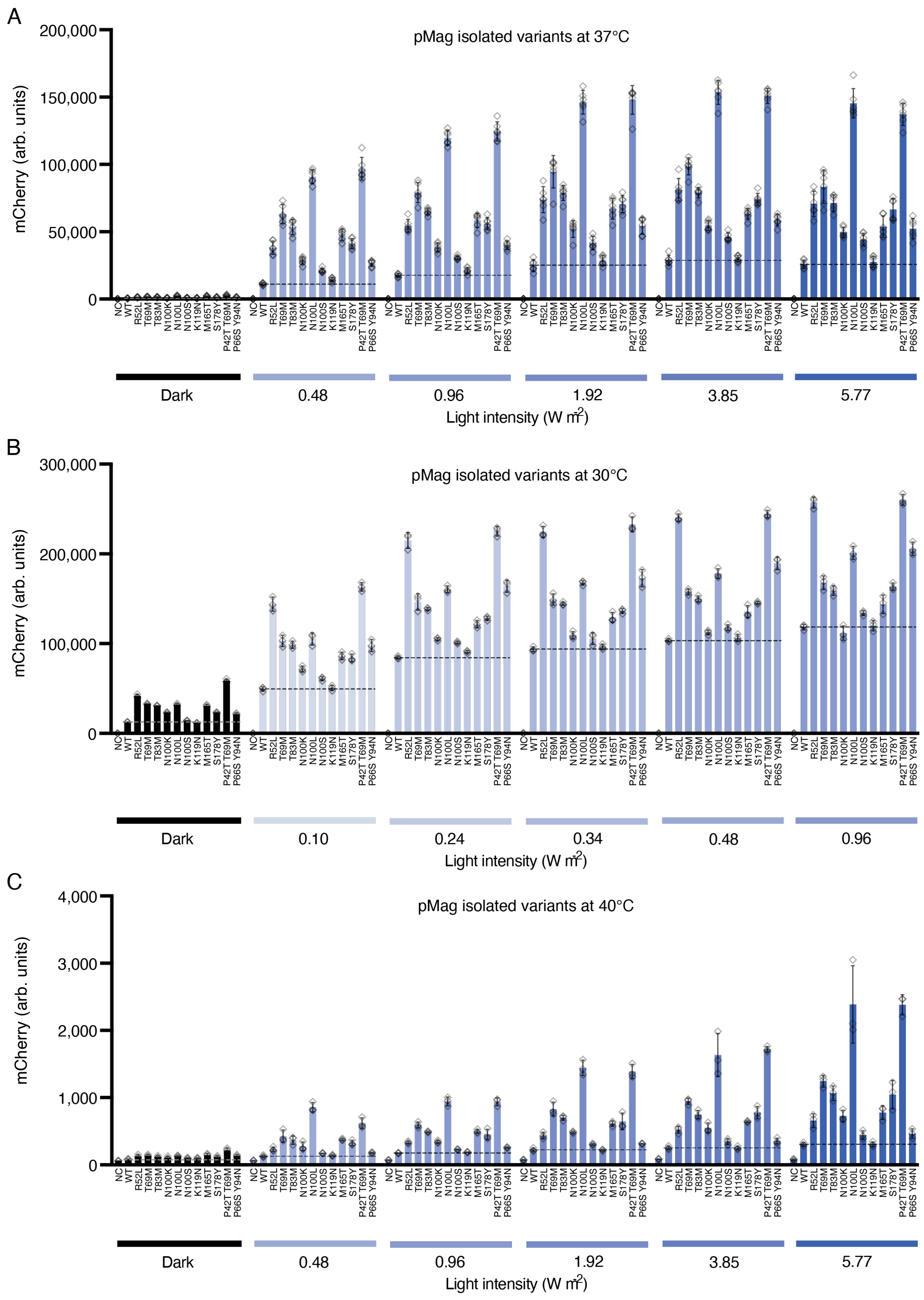

Supplementary Figure 4: Characterization of identified mutations in pMag incubated at 37°C (A), 30°C (B) and 40°C (C) through flow cytometry in comparison to the wild-type Opto-T7RNAP*(563) regulator and a negative control (NC) containing the mCherry expression plasmid and a second empty plasmid that does not contain the optogenetic regulator. mCherry expression values were acquired after 5h incubation at the indicated light intensity and temperature. Shown are the mean fluorescence values and standard deviation (mean values +/- SD) as well as individual data points of six (n=6) biological replicates for (A) and three (n=3) biological replicates for (B) and (C).

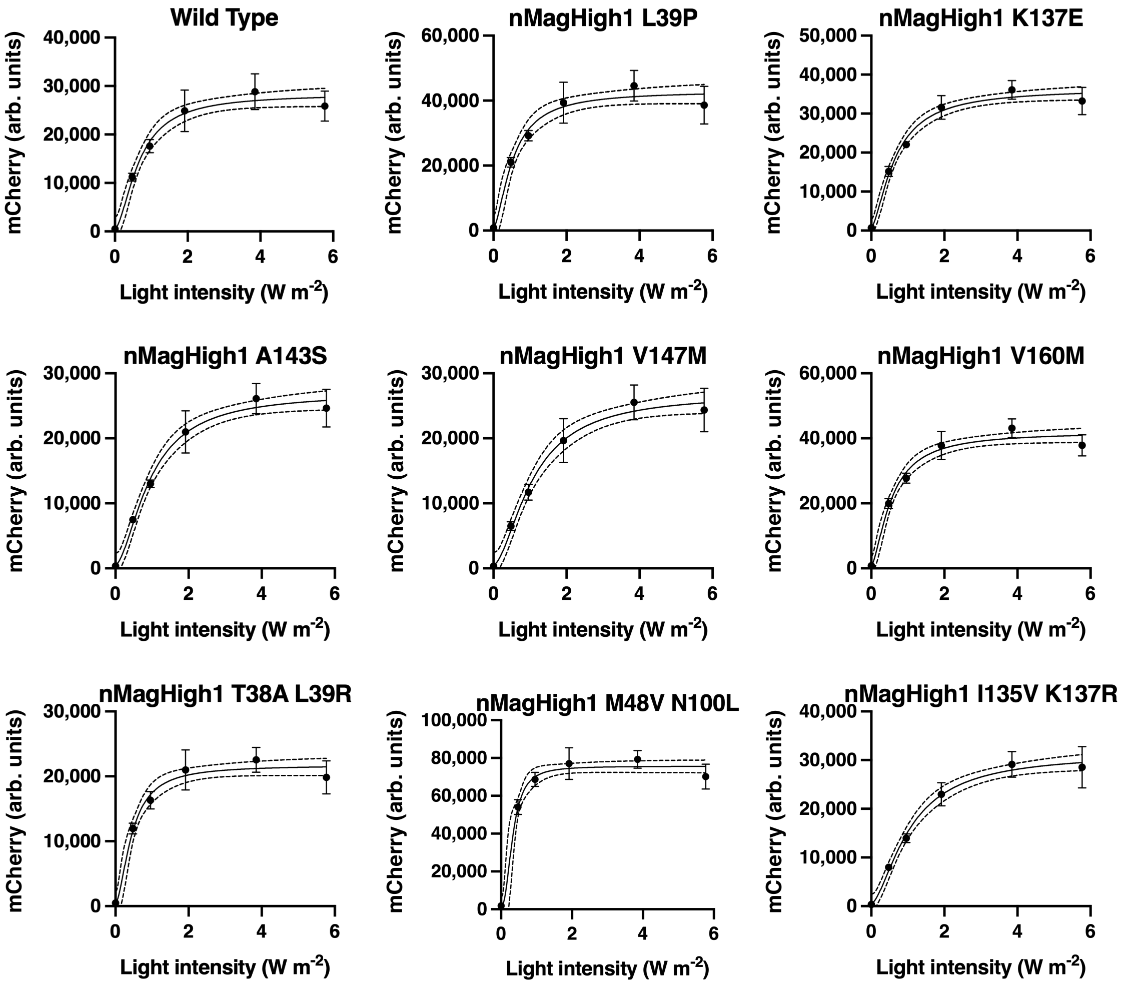

Supplementary Figure 5: Dose-response curves of mCherry fluorescence of Opto-T7RNAP*(563) and different nMagHigh1 variants in response to varying light intensities. Cultures were incubated for 5h at 37°C and endpoints measured through flow cytometry. Shown are mean and standard deviation (mean values +/- SD) of six biological replicates (n=6) and mathematical fits (solid lines) with lower and upper 95% profile-likelihood confidence limits (dashed lines).

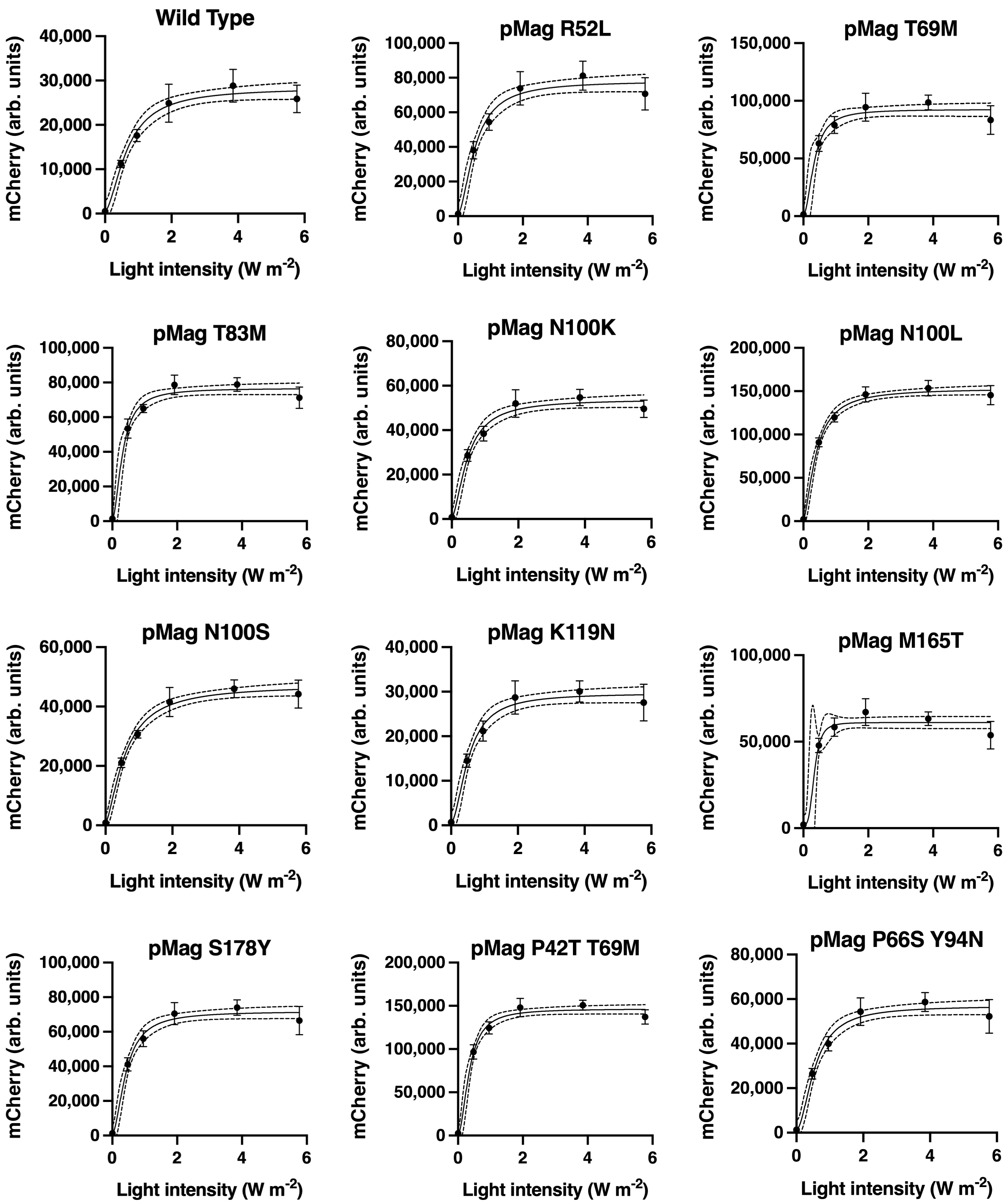

Supplementary Figure 6: Dose-response curves of mCherry fluorescence of Opto-T7RNAP*(563) and different pMag variants in response to varying light intensities. Cultures were incubated for 5h at 37°C and endpoints measured through flow cytometry. Shown are mean and standard deviation (mean values +/- SD) of six biological replicates (n=6) and mathematical fits (solid lines) with lower and upper 95% profile-likelihood confidence limits (dashed lines).

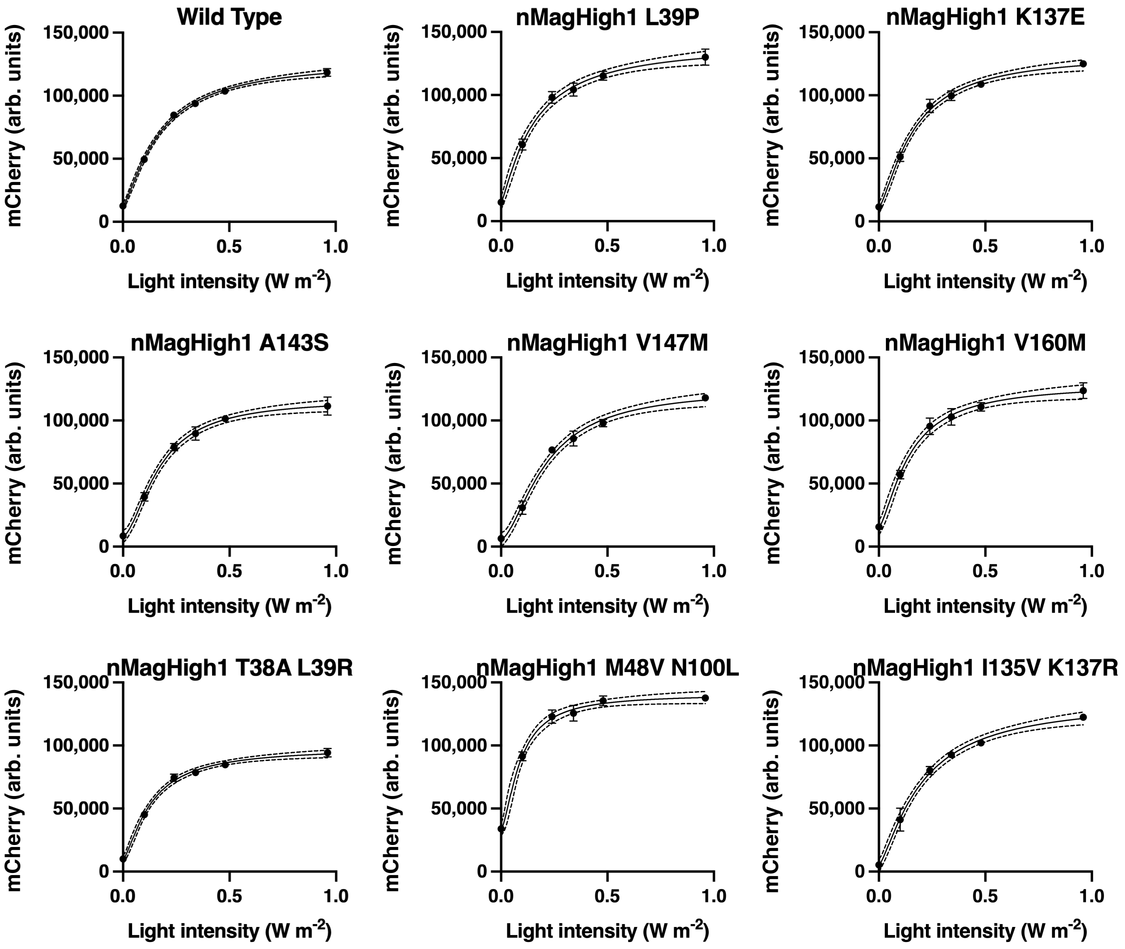

Supplementary Figure 7: Dose-response curves of mCherry fluorescence of Opto-T7RNAP*(563) and different nMagHigh1 variants in response to varying light-intensities. Cultures were incubated for 5h at 30°C and endpoints measures through flow cytometry. Shown are mean and standard deviation (mean values +/- SD) of three biological replicates (n=3) and mathematical fits (solid lines) with lower and upper 95% profile-likelihood confidence limits (dashed lines).

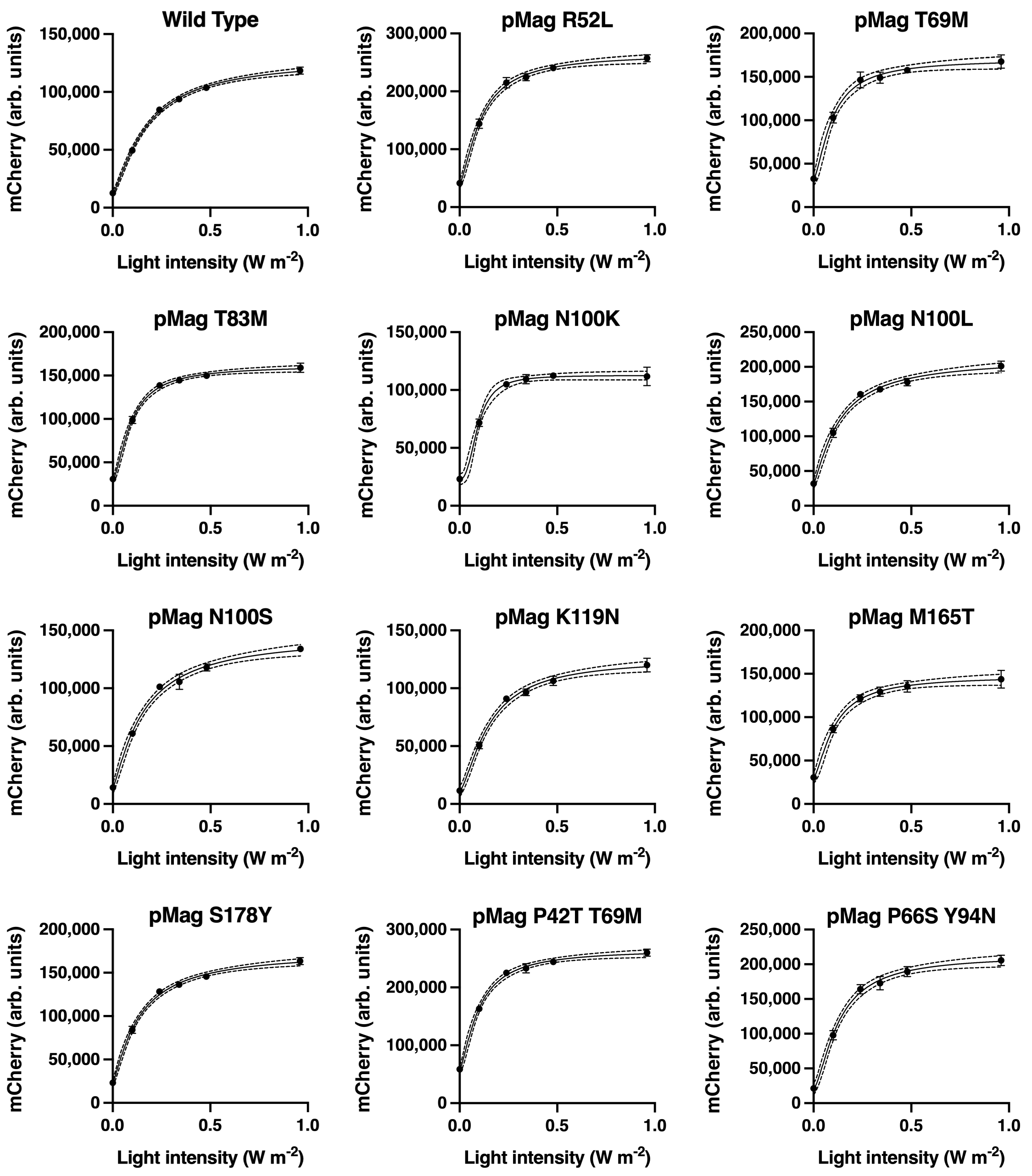

Supplementary Figure 8: Dose-response curves of mCherry fluorescence in response to varying light-intensity of Opto-T7RNAP*(563) and different pMag variants. Cultures were incubated for 5h at 30°C and endpoints measures through flow cytometry. Shown are mean and standard deviation (mean values +/- SD) of three biological replicates (n=3) and mathematical fits (solid lines) with lower and upper 95% profile-likelihood confidence limits (dashed lines).

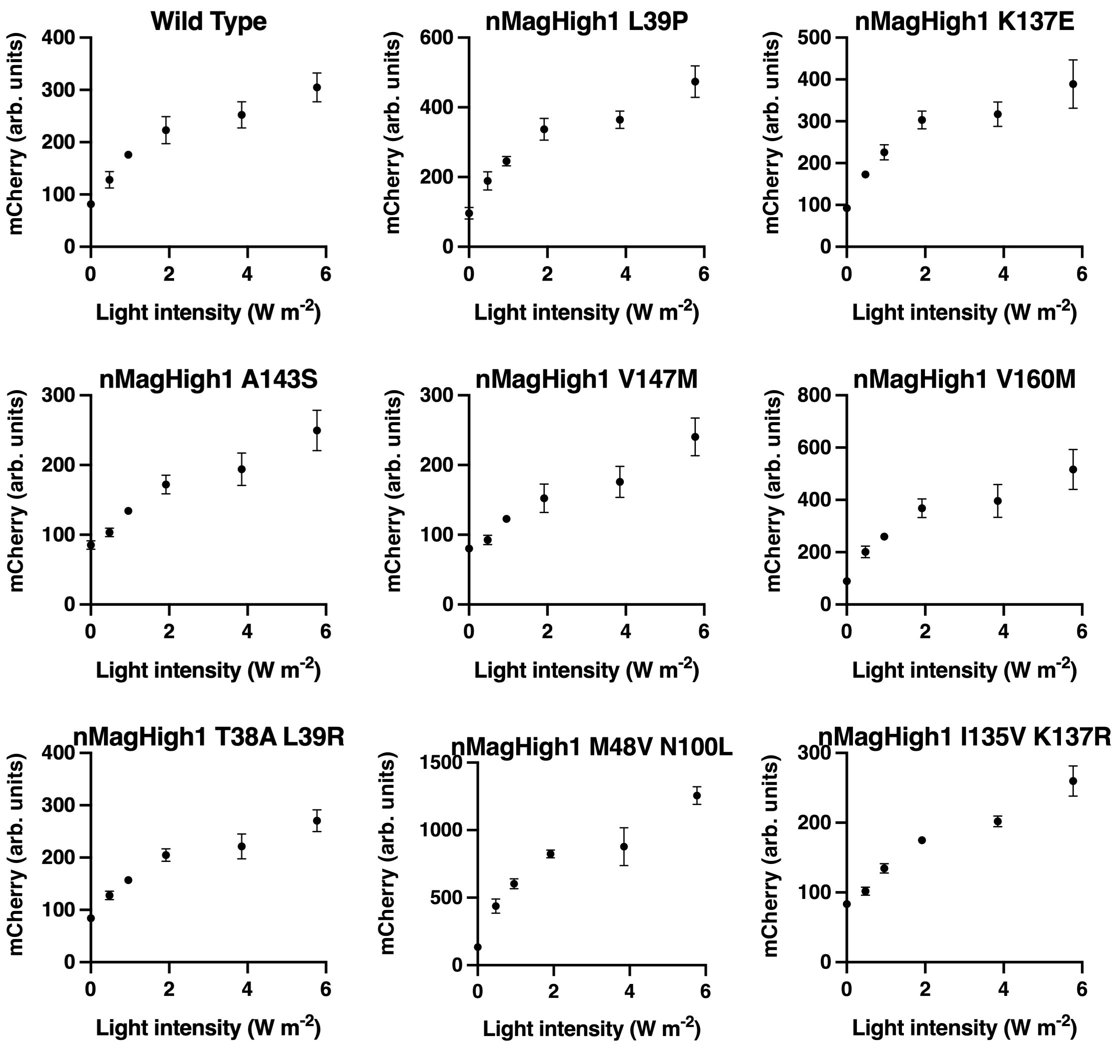

Supplementary Figure 9: Dose-response curves of mCherry fluorescence in response to varying light-intensity of Opto-T7RNAP*(563) and different nMagHigh1 variants. Cultures were incubated for 5h at 40°C and endpoints measured through flow cytometry. Shown are mean and standard deviation (mean values +/- SD) of three biological replicates (n=3).

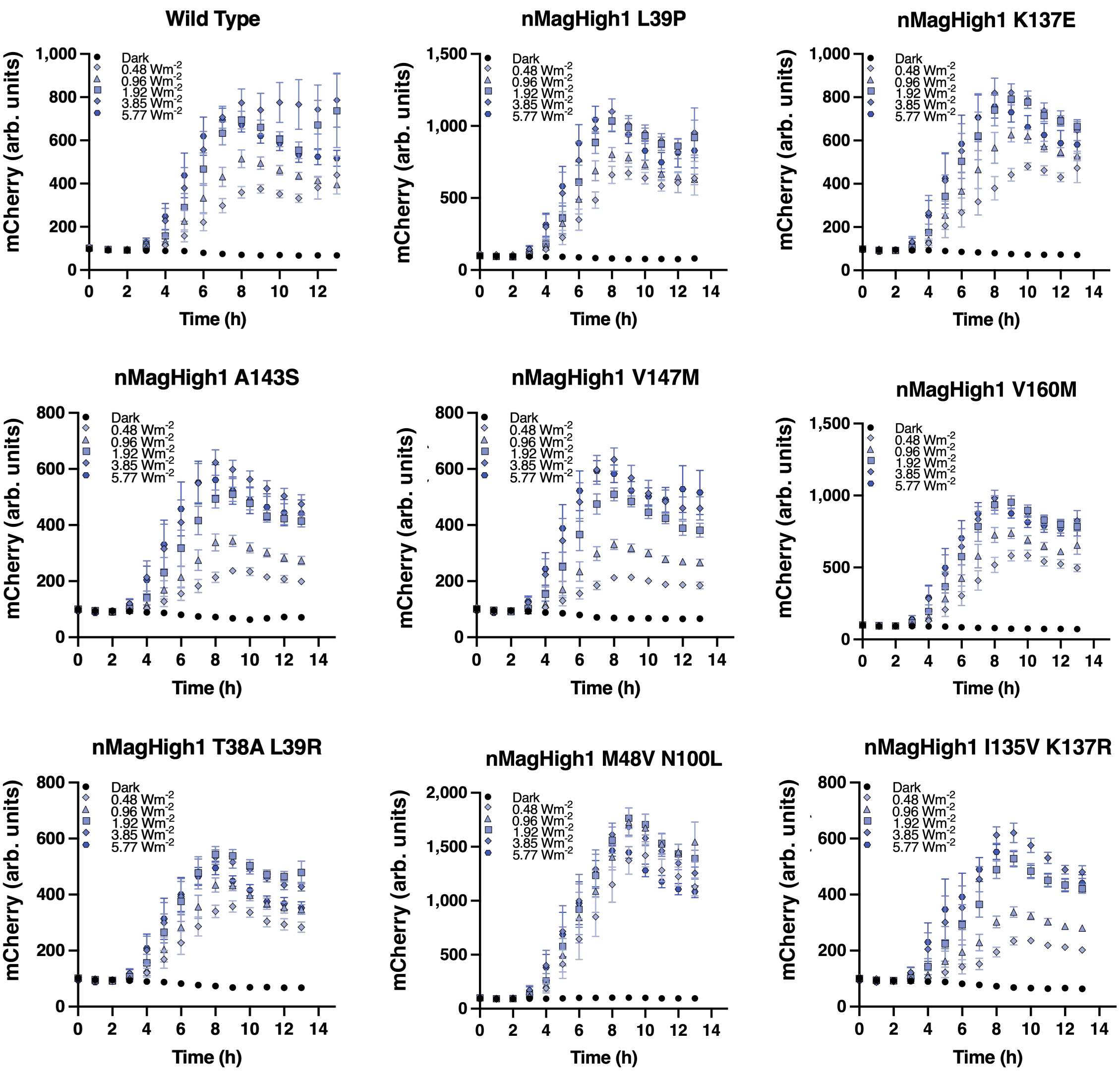

Supplementary Figure 10: Expression of mCherry fluorescence measured through spectrophotometry over time of Opto-T7RNAP*(563) and different nMagHigh1 variants in response to varying light intensities. Cultures were incubated at 37°C. Shown are mean and standard error of the mean fluorescence values of at least six (n=6-9) biological replicates.

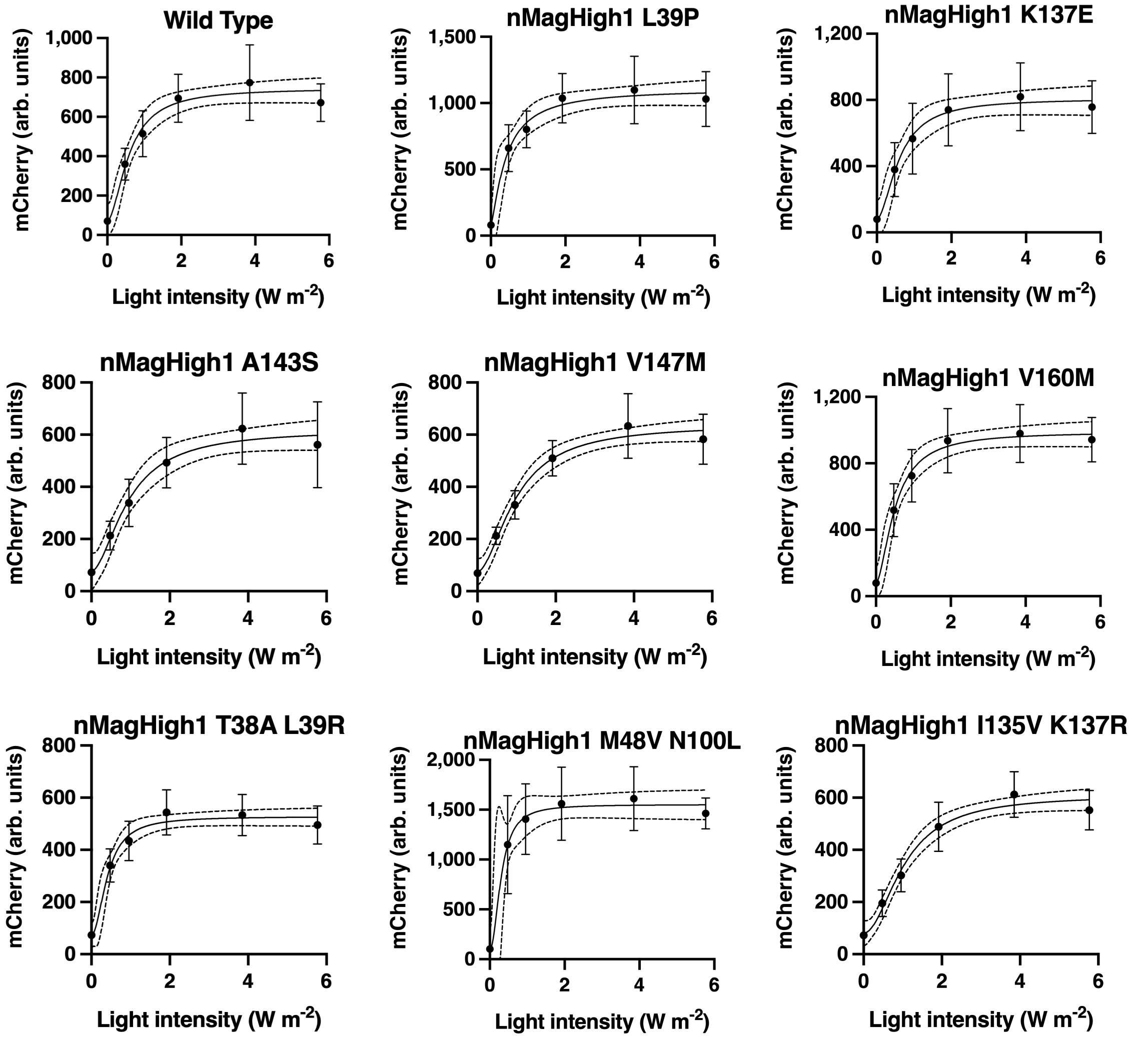

Supplementary Figure 11: Expression of mCherry fluorescence measured through spectrophotometry at timepoint 8h of Opto-T7RNAP*(563) and different nMagHigh1 variants in response to varying light intensities. Cultures were incubated at 37°C. Shown are mean and standard error of the mean fluorescence values at least six (n=6-9) biological replicates.

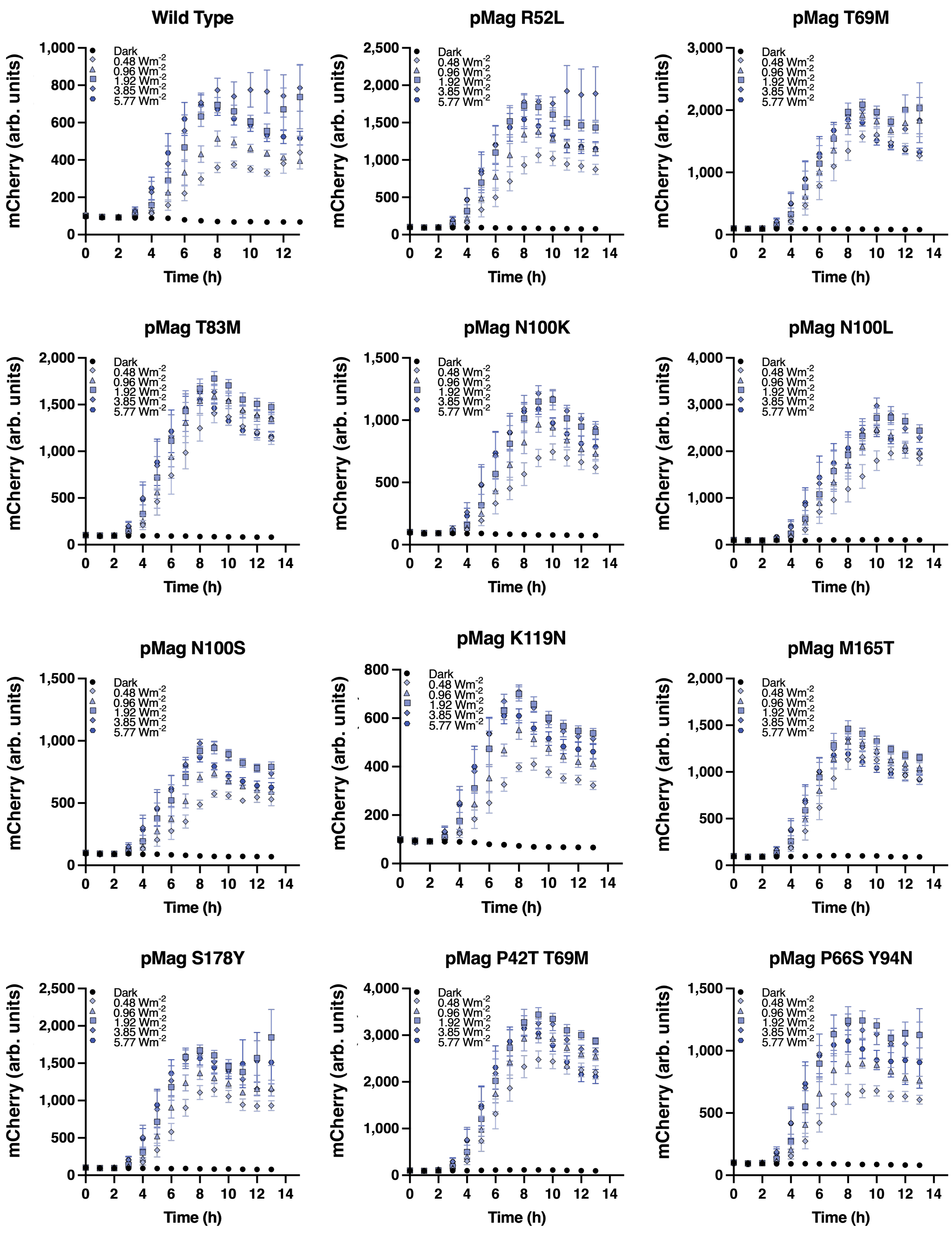

Supplementary Figure 12: Expression of mCherry fluorescence measured through spectrophotometry over time of Opto-T7RNAP*(563) and different pMag variants in response to varying light-intensities. Cultures were incubated at 37°C. Shown are mean and standard error of the mean fluorescence values of at least six (n=6-9) biological replicates.

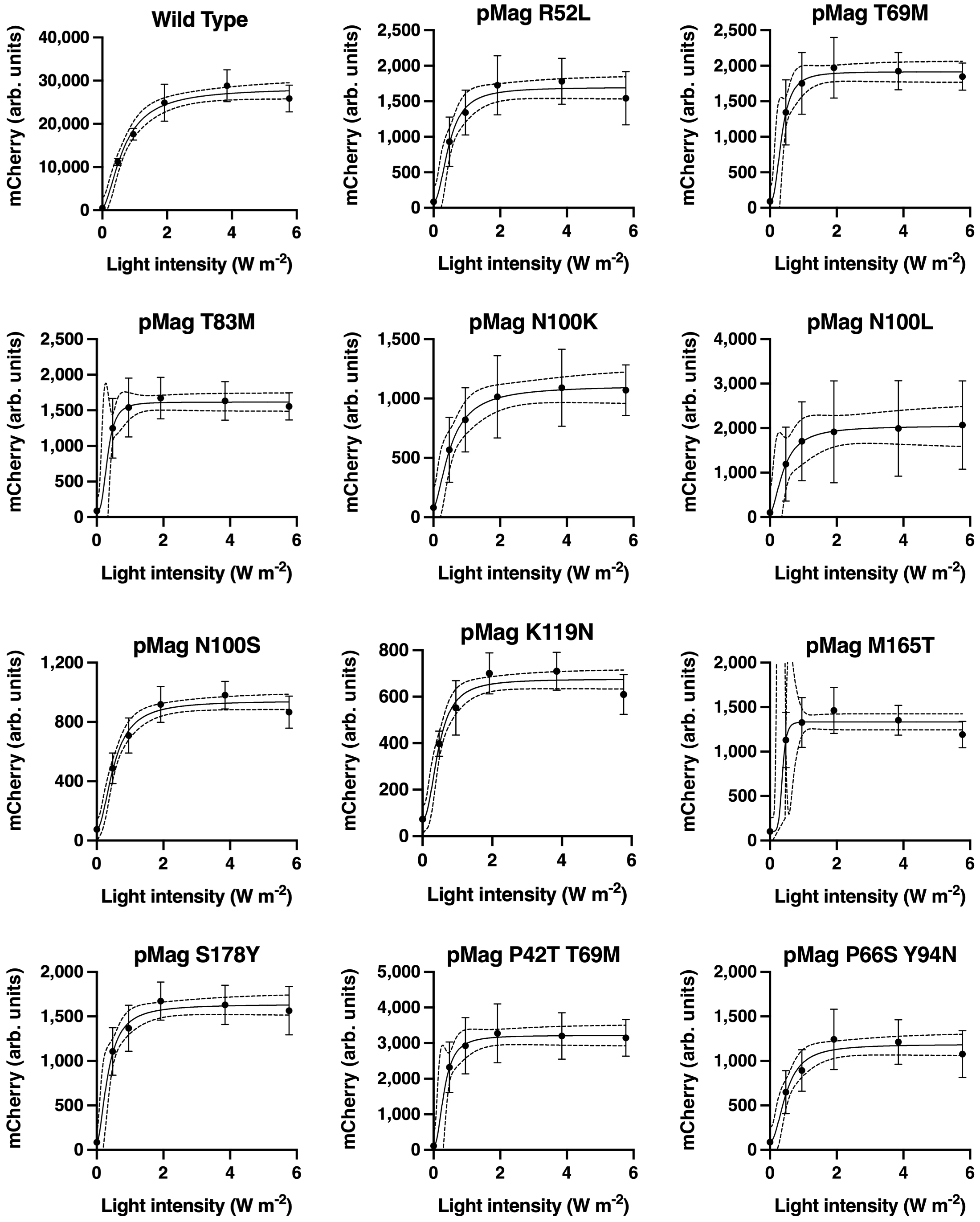

Supplementary Figure 13: Expression of mCherry fluorescence measured through spectrophotometry at timepoint 8h of Opto-T7RNAP*(563) and different nMagHigh1 variants in response to varying light-intensities. Cultures were incubated at 37°C. Shown are mean fluorescence values of of at least six (n=6-9) biological replicates.

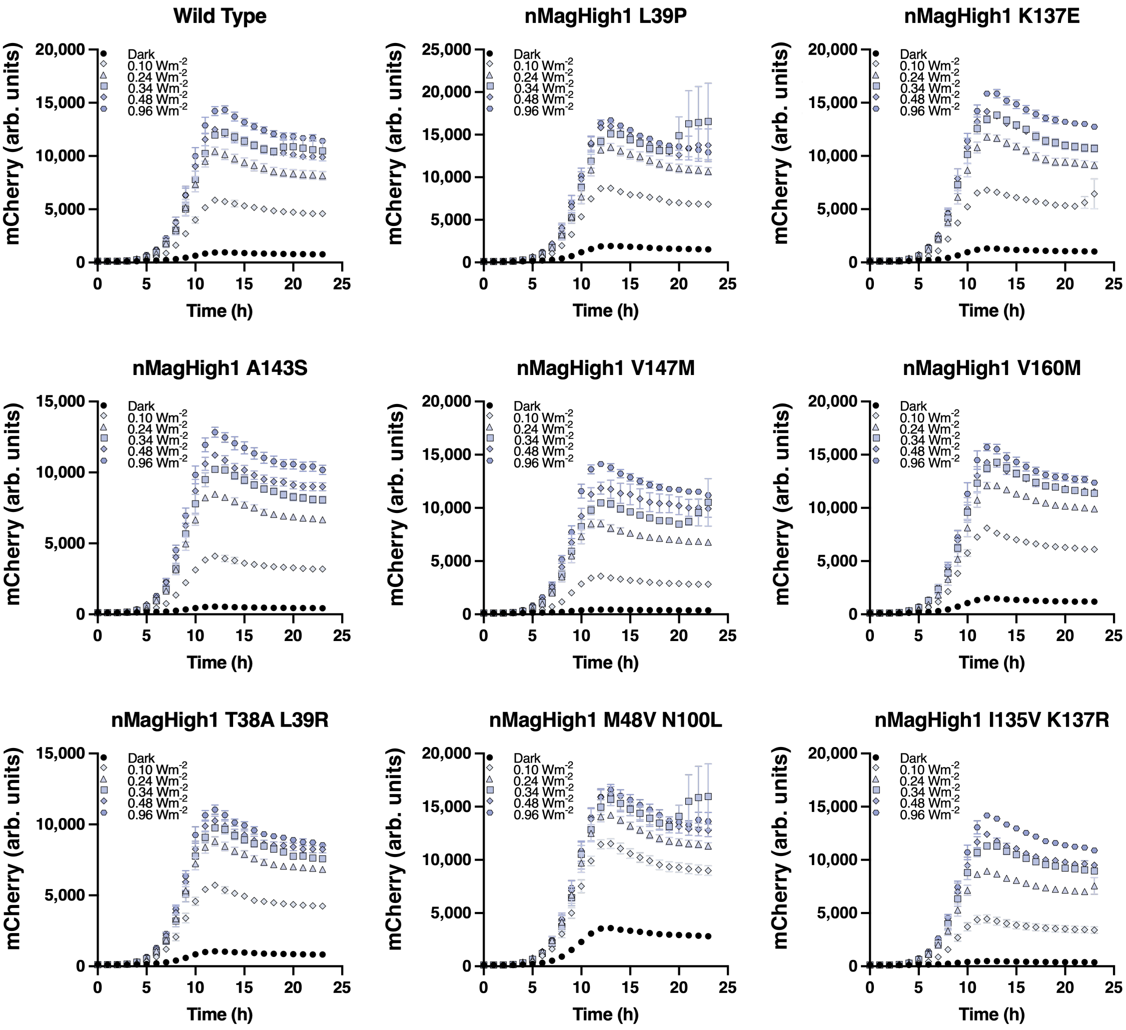

Supplementary Figure 14: Expression of mCherry fluorescence measured through spectrophotometry over time of Opto-T7RNAP*(563) and different nMagHigh1 variants in response to varying light-intensities. Cultures were incubated at 30°C. Shown are mean and standard error of the mean fluorescence values of at least six (n=6-9) biological replicates.

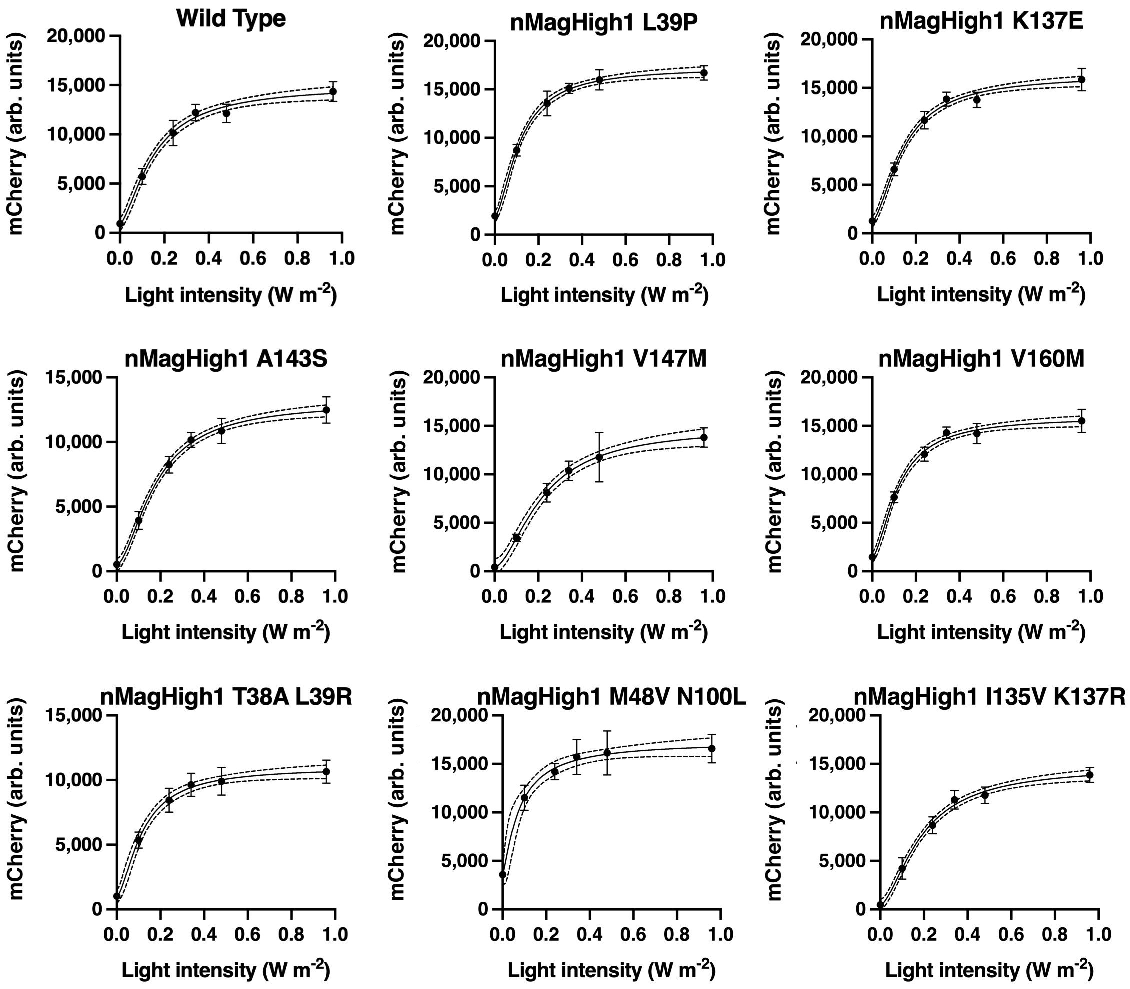

Supplementary Figure 15: Expression of mCherry fluorescence measured through spectrophotometry at timepoint 13h of Opto-T7RNAP*(563) and different nMagHigh1 variants in response to varying light-intensities. Cultures were incubated at 30°C. Shown are mean and standard error of the mean fluorescence values of at least six (n=6-9) biological replicates.

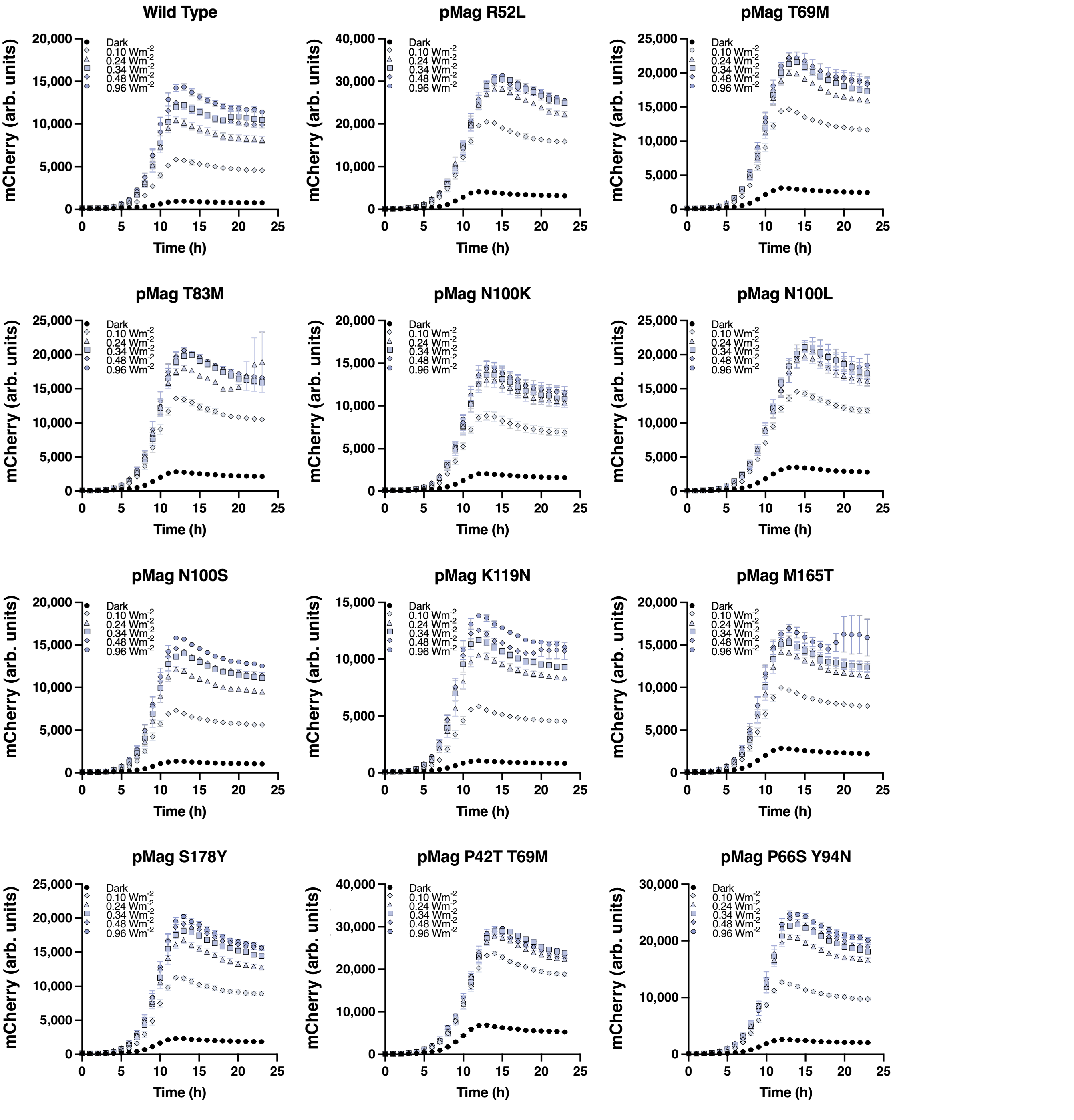

Supplementary Figure 16: Expression of mCherry fluorescence measured through spectrophotometry over time in response to varying light-intensity of Opto-T7RNAP*(563) and different pMag variants. Cultures were incubated at 30°C. Shown are mean and standard error of the mean fluorescence values of at least six (n=6-9) biological replicates.

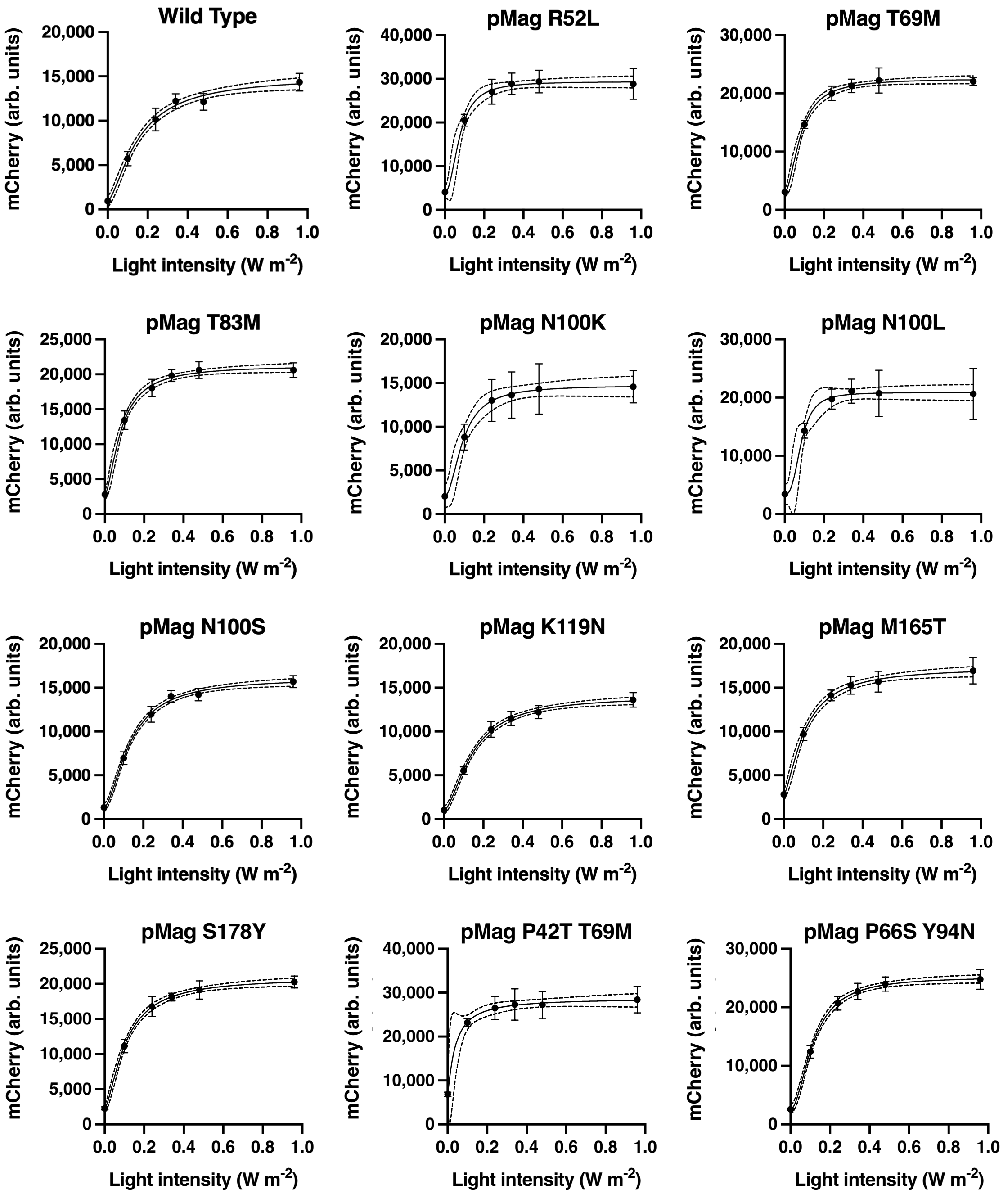

Supplementary Figure 17: Expression of mCherry fluorescence measured through spectrophotometry at timepoint 13h in response to varying light-intensity of Opto-T7RNAP*(563) and different pMag variants and at timepoint 15h for pMag N100L due to the later expression (see Supplementary Figure 16). Cultures were incubated at 30°C. Shown are mean fluorescence values of at least six biological replicates (n=6-9).

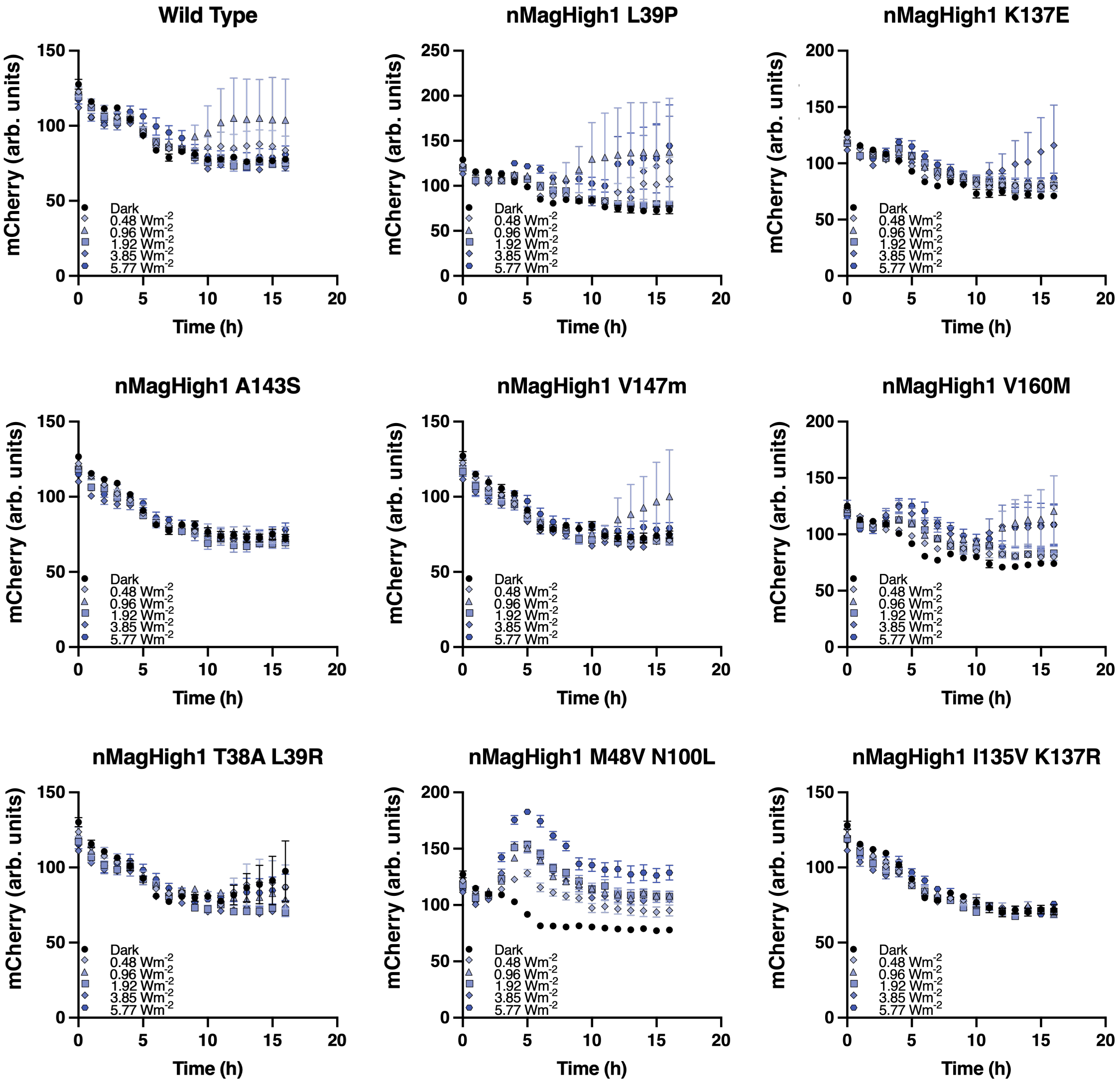

Supplementary Figure 18: Expression of mCherry fluorescence measured through spectrophotometry over time in response to varying light-intensity of Opto-T7RNAP*(563) and different nMagHigh1Mag variants. Cultures were incubated at 40°C. Shown are mean and standard error of the mean fluorescence values of six (n=6) biological replicates.

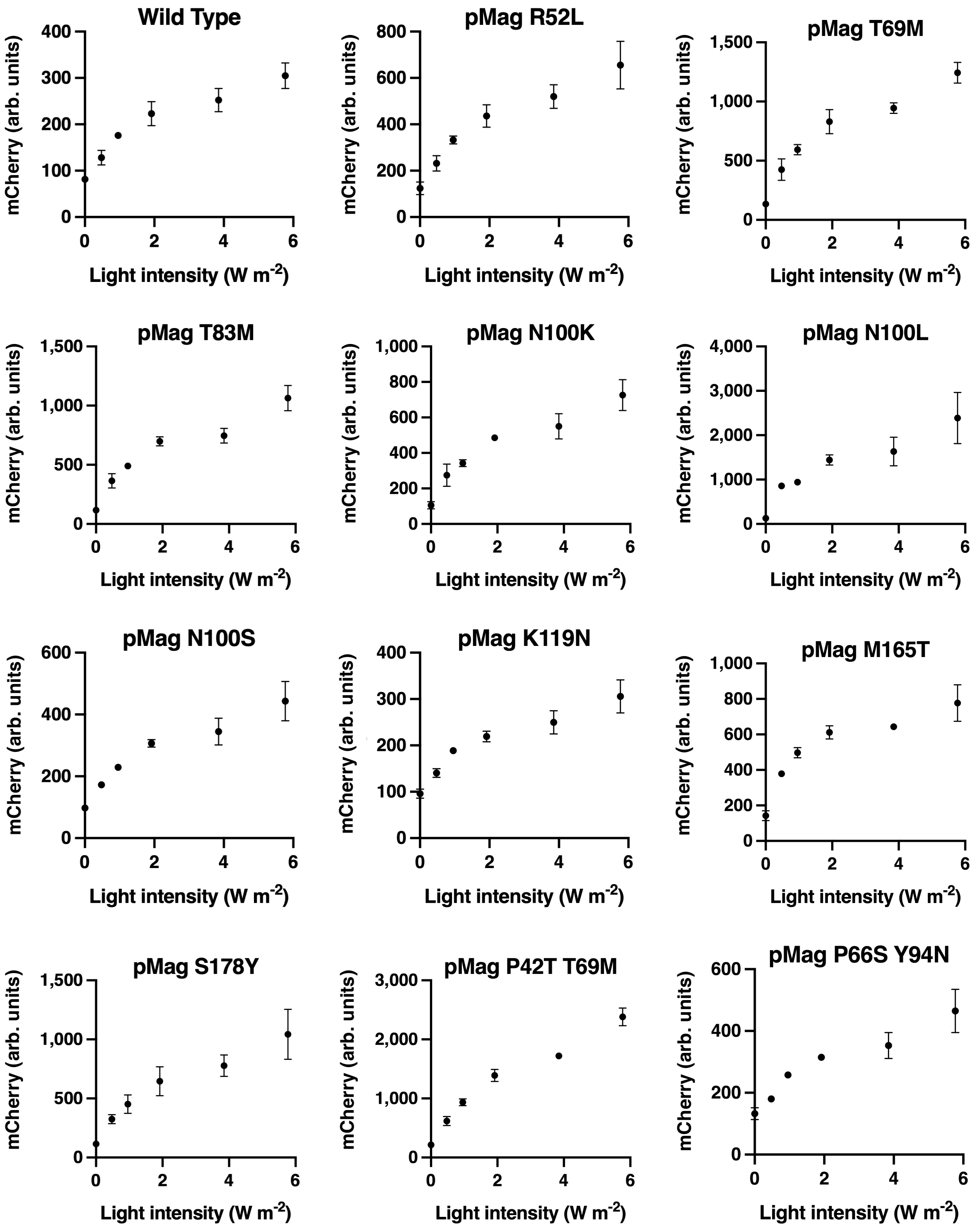

Supplementary Figure 19: Dose-response curves of mCherry fluorescence in response to varying light-intensity of Opto-T7RNAP*(563) and different pMag variants. Cultures were incubated for 5h at 40°C and endpoints measures through flow cytometry. Shown are mean and standard deviation (mean values +/- SD) of three biological replicates (n=3).

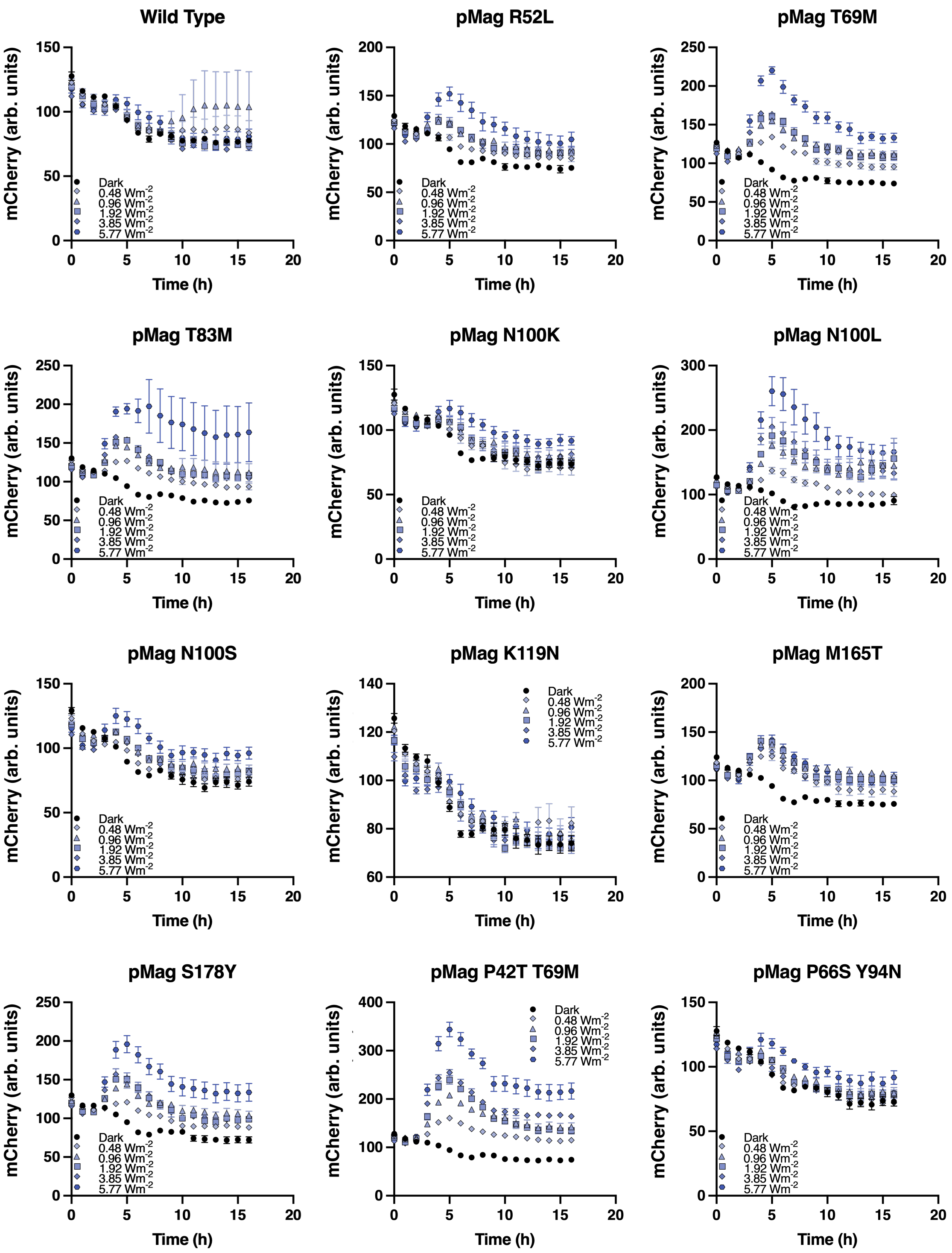

Supplementary Figure 20: Expression of mCherry fluorescence measured through spectrophotometry over time in response to varying light-intensity of Opto-T7RNAP*(563) and different pMag variants. Cultures were incubated at 40°C. Shown are mean and standard error of the mean fluorescence values of six (n=6) biological replicates.

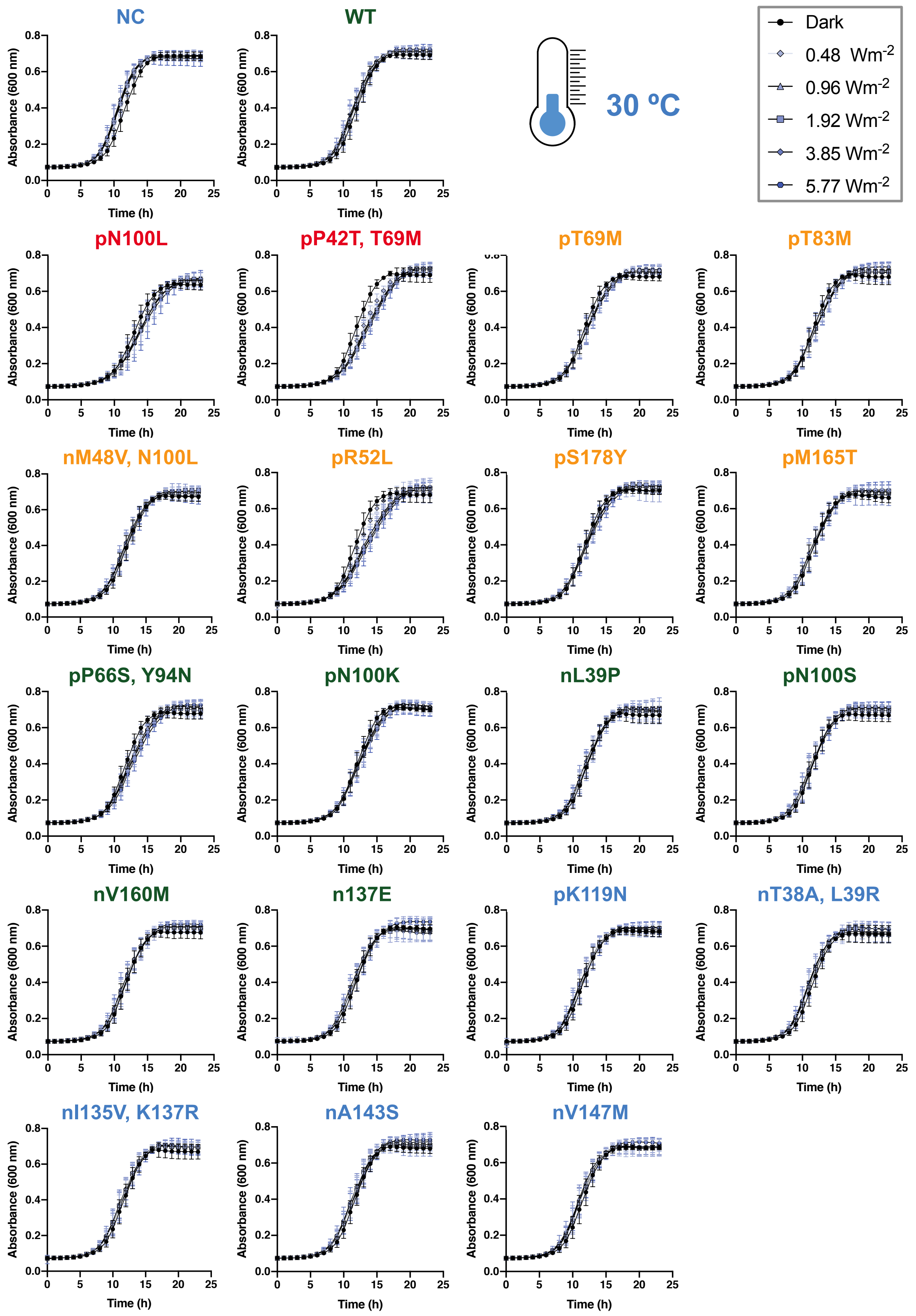

Supplementary Figure 21: Bacterial growth measured through absorbance at 600 nm over time in response to varying light-intensity of Opto-T7RNAP*(563) and different nMagHigh1 and pMag variants. Growth data corresponds to the respective variants shown in Supplementary Figure 14 and Supplementary Figure 16. Cultures were incubated at 30°C. Shown are mean and standard error of absorbance values at 600 nm of at least six (n=6-9) biological replicates.

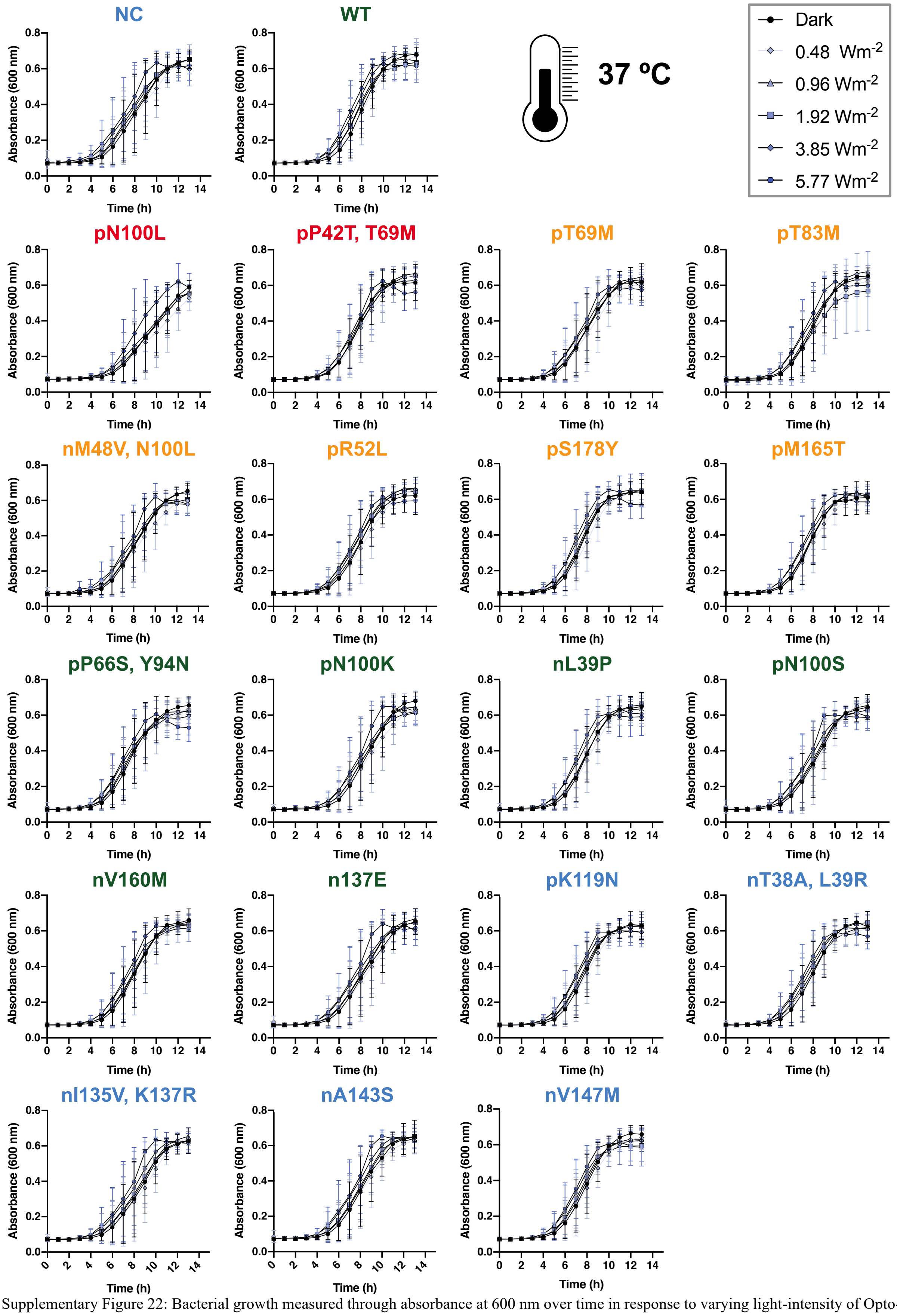

Supplementary Figure 22: Bacterial growth measured through absorbance at 600 nm over time in response to varying light-intensity of Opto-T7RNAP*(563) and different nMagHigh1 and pMag variants. Growth data corresponds to the respective variants shown in Supplementary Figure 10 and Supplementary Figure 12. Cultures were incubated at 37°C. Shown are mean and standard error of absorbance values at 600 nm of at least six (n=6-9) biological replicates.

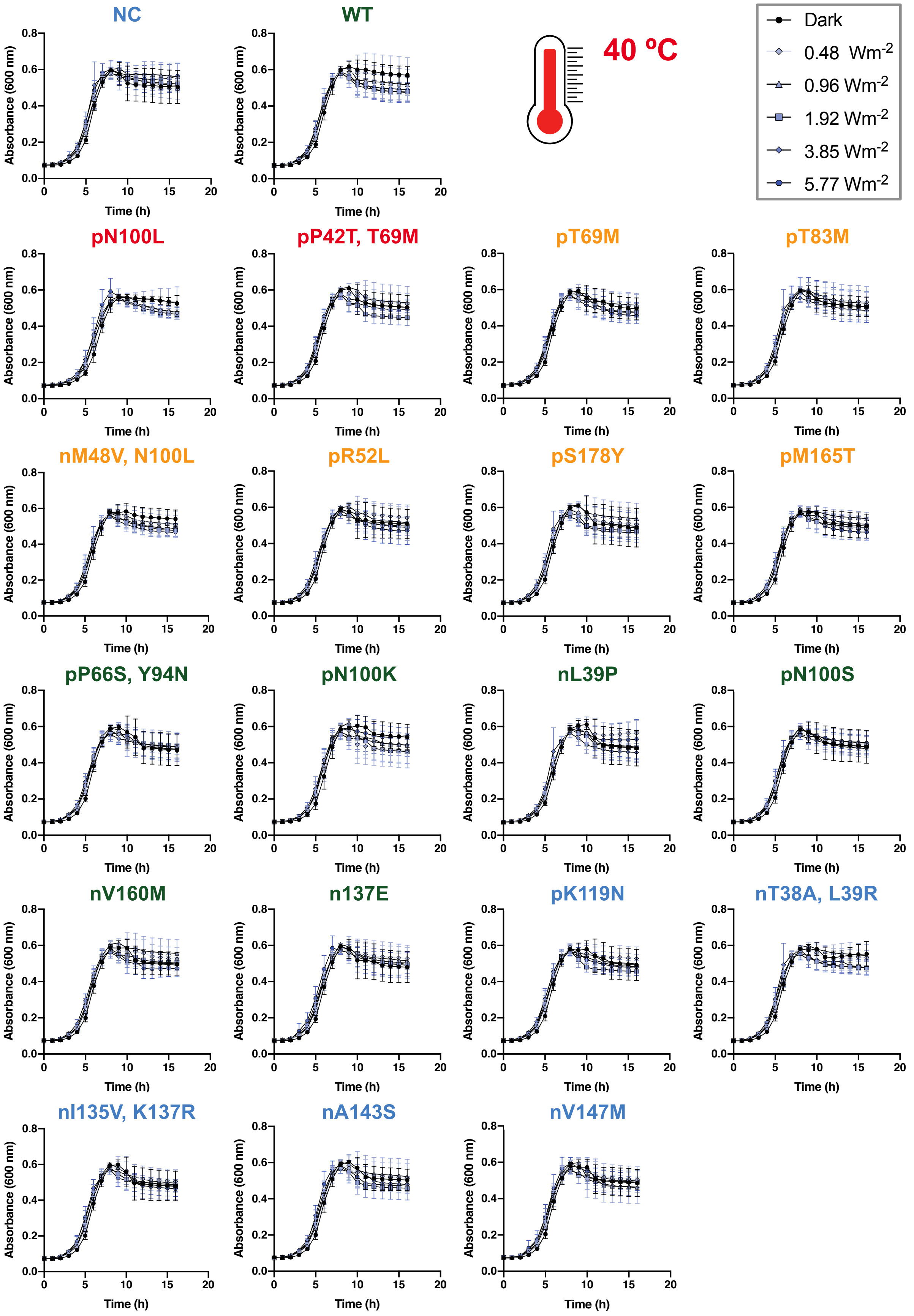

Supplementary Figure 23: Bacterial growth measured through absorbance at 600 nm over time in response to varying light-intensity of Opto-T7RNAP*(563) and different nMagHigh1 and pMag variants. Growth data corresponds to the respective variants shown in Supplementary Figure 18 and Supplementary Figure 20. Cultures were incubated at 37°C. Shown are mean and standard error of absorbance values at 600 nm of six (n=6) biological replicates.

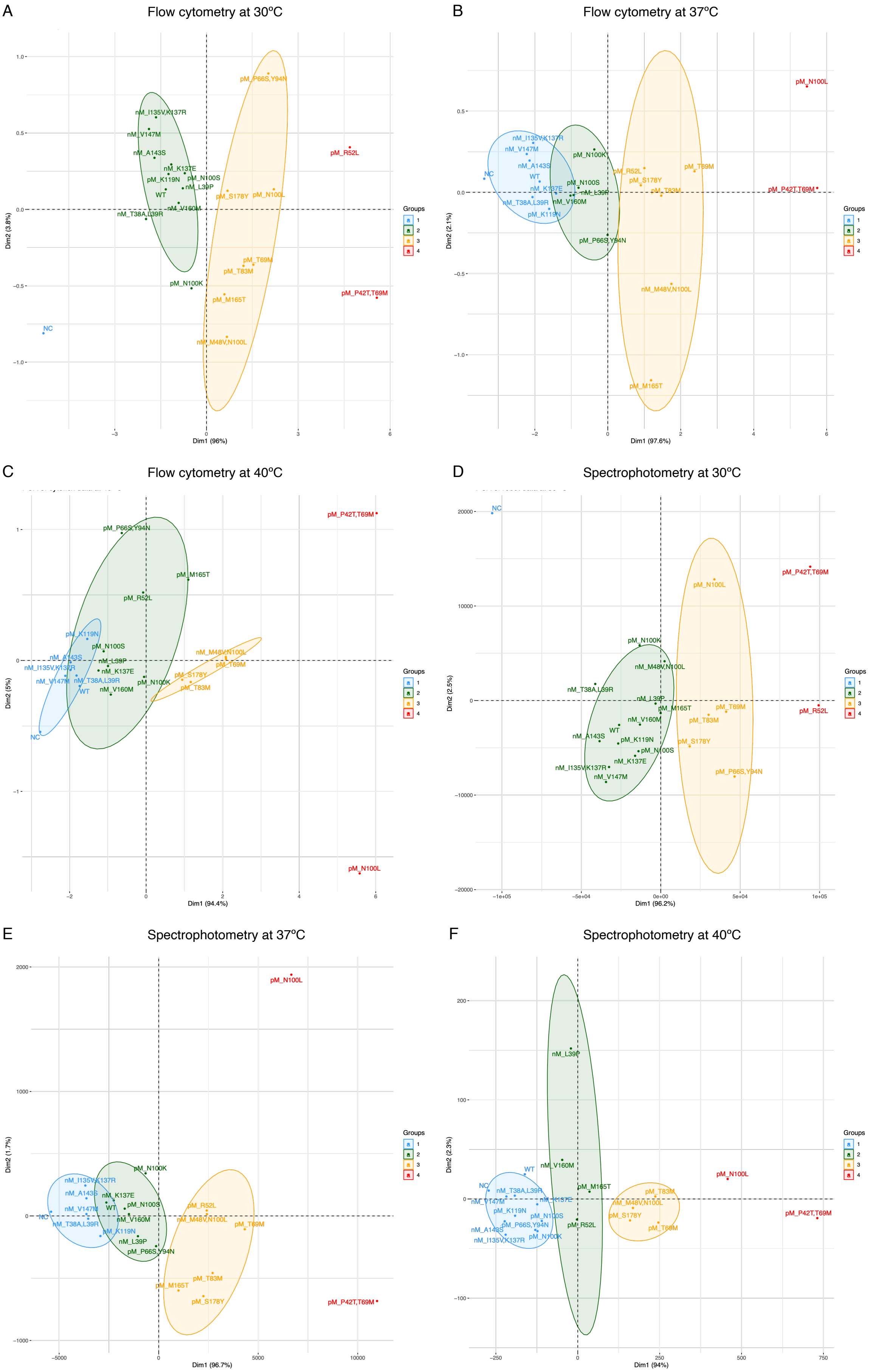
A
Flow cytometry at 30°C
Dim1 (96%)
Dim2 (3.8%)
nM_I135V,K137R
nM_V147M
nM_A143S
nM_K137E
pM_K119N
pM_N100S
nM_L39P
WT
nM_V160M
nM_T38A,L39R
pM_P66S,Y94N
pM_S178Y
pM_N100L
pM_R52L
pM_T69M
pM_T83M
pM_N100K
pM_M165T
nM_M48V,N100L
pM_P42T,T69M
NC
Groups
1
2
3
4
B
Flow cytometry at 37°C
Dim1 (97.6%)
Dim2 (2.1%)
pM_N100L
nM_I135V,K137R
nM_V147M
nM_A143S
NC
WT
nM_K137E
nM_T38A,L39R
pM_K119N
pM_N100K
pM_N100S
nM_L39P
nM_V160M
pM_P66S,Y94N
pM_R52L
pM_S178Y
pM_T69M
pM_T83M
pM_P42T,T69M
nM_M48V,N100L
pM_M165T
Groups
1
2
3
4
C
Flow cytometry at 40°C
Dim1 (94.4%)
Dim2 (5%)
pM_P42T,T69M
pM_P66S,Y94N
pM_M165T
pM_R52L
pM_K119N
pM_N100S
nM_A143S
nM_I135V,K137R
nM_L39P
nM_K137E
nM_V147M
nM_T38A,L39R
WT
nM_V160M
pM_N100K
pM_S178Y
pM_T83M
nM_M48V,N100L
pM_T69M
NC
pM_N100L
Groups
1
2
3
4
D
Spectrophotometry at 30°C
Dim1 (96.2%)
Dim2 (2.5%)
NC
pM_P42T,T69M
pM_N100L
pM_N100K
nM_M48V,N100L
nM_T38A,L39R
nM_L39P
pM_M165T
pM_T69M
pM_R52L
pM_T83M
nM_V160M
WT
nM_A143S
pM_K119N
pM_N100S
pM_S178Y
nM_K137E
nM_I135V,K137R
nM_V147M
pM_P66S,Y94N
Groups
1
2
3
4
E
Spectrophotometry at 37°C
Dim1 (96.7%)
Dim2 (1.7%)
pM_N100L
pM_N100K
nM_I135V,K137R
nM_A143S
nM_K137E
nM_V147M
WT
pM_N100S
NC
nM_V160M
nM_T38A,L39R
pM_K119N
nM_L39P
pM_P66S,Y94N
pM_R52L
nM_M48V,N100L
pM_T69M
pM_T83M
pM_M165T
pM_S178Y
pM_P42T,T69M
Groups
1
2
3
4
F
Spectrophotometry at 40°C
Dim1 (94%)
Dim2 (2.3%)
nM_L39P
nM_V160M
WT
NC
nM_T38A,L39R
nM_V147M
nM_K137E
pM_M165T
pM_K119N
pM_N100S
pM_P66S,Y94N
nM_A143S
pM_R52L
nM_I135V,K137R
pM_N100K
pM_T83M
nM_M48V,N100L
pM_S178Y
pM_T69M
pM_N100L
pM_P42T,T69M
Groups
1
2
3
4

Supplementary Figure 24: PC analysis of the single-cell expression data (flow cytometry; A-C) as well as population data (spectrophotometry; D-F) at the indicated temperatures. (A-C) Cultures were incubated for 5h and mCherry fluorescence at endpoints was measured by flow cytometry (Supplementary Figures 3-9). (D-F) Expression of mCherry fluorescence was measured over time by spectrophotometry (Supplementary Figures 10-20). X-axis shows that PC1 explains 96 %, 97.6% and 94.4% (A-C) and 96.2%, 96.7% and 94% (D-F) of the differences at 30, 37 and 40 ºC, respectively. Mutant variants are colored according to the clusters selected by hierarchical clustering (see Figure 3 E-F). Red: very high expression; Orange: high expression; Green: medium expression; Blue: low expression.

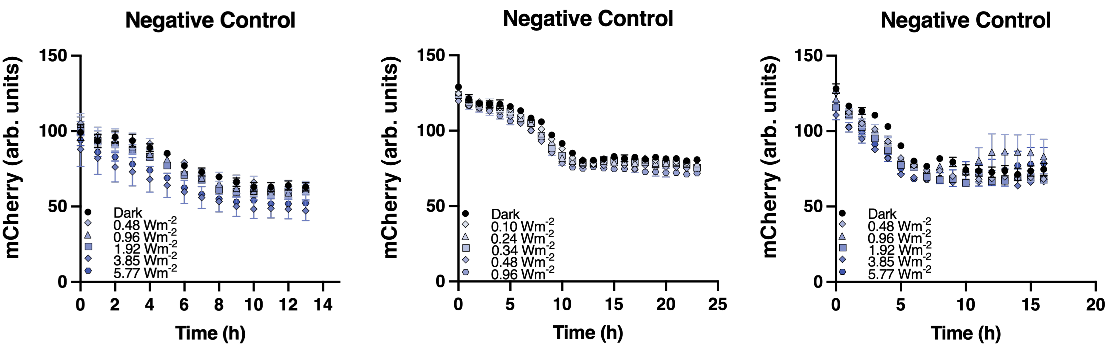

Supplementary Figure 25: Expression of mCherry fluorescence measured through spectrophotometry over time in response to varying light intensity of negative controls. Cultures were incubated at 37°C (left), 30°C (middle) and 40°C (right). Shown are mean and standard error of the mean fluorescence values of at least six (n=6-9) biological replicates.

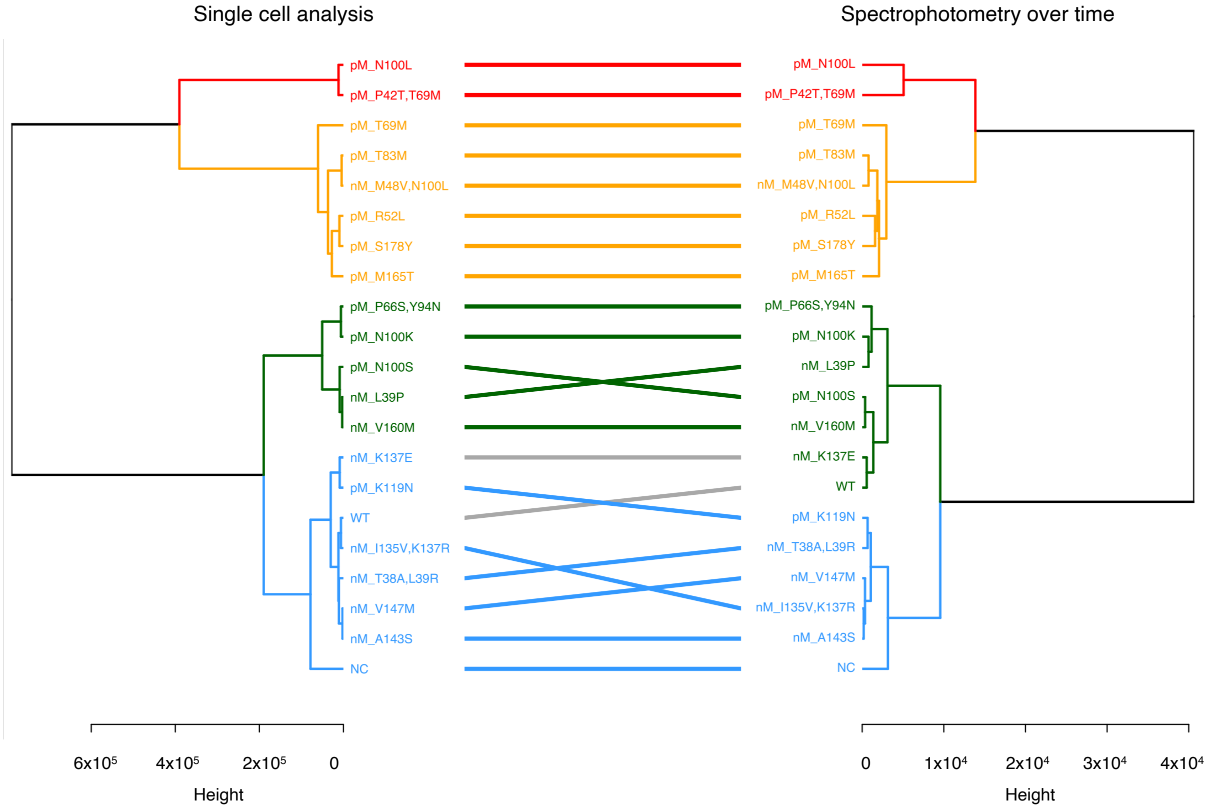

Supplementary Figure 26: Comparison of the dendrograms depicting the hierarchical clustering of variants according to the expression levels at 37ºC for the single cell data (left clustering) and spectrophotometry data (right clustering). Colored horizontal lines connect variants in the same expression clusters in both types of data. Grey horizontal lines connect variants located in different expression clusters in both types of data (i.e. WT and nMag K137E). Red: very high expression; Orange: high expression; Green: medium expression; Blue: low expression.

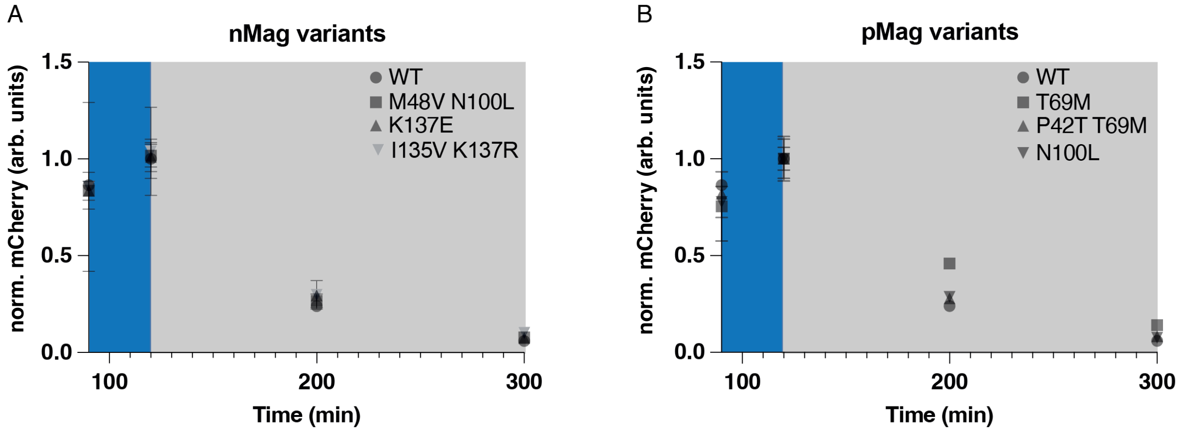

Supplementary Figure 27: Time course of selected variants. (A) nMagHigh1 variants M48V N100L, K137E, and I135V 137R in comparison to the wild-type Opto-T7RNAP*(563). (B) pMag variants T69M, P41T T69M, and N100L in comparison to the wild-type Opto-T7RNAP*(563). The samples were induced for 120 min at saturating light intensity (3.85 W m^{-2}) before switching the light off at timepoint 120 min. For each variant, the normalization was calculated by dividing the respective mCherry expression level by the mean mCherry expression level of all replicates at timepoint 120 min. All variants show the highest mCherry fluorescence at 120 minutes. To allow for comparison of variants with different mCherry expression levels, mCherry expression of all variants were normalized to their respective highest mean mCherry expression at 120 min. The diagram shows normalized mean mCherry expression values and standard deviation (mean values +/- SD) of three biological replicates (n=3) measured after 5h incubation time.

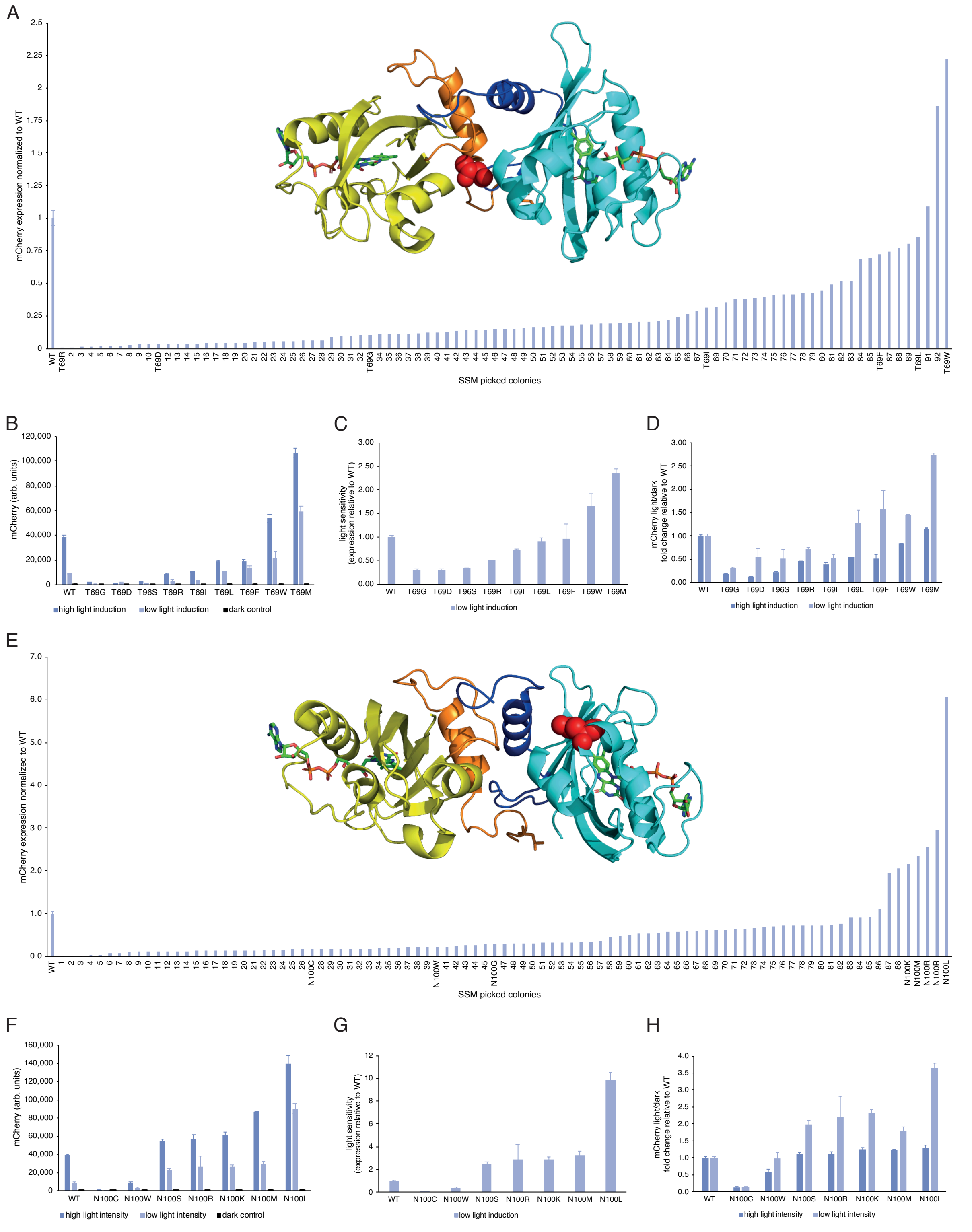

Supplementary Figure 28: Site Saturation Mutagenesis (SSM) of positions T69 and N100 in pMag. (A) mCherry expression level of 93 variants obtained through SSM at position (A) T69 or (E) N100. Variants with various expression levels as well as with increased expression compared to the WT were sequenced and annotated. The inset shows the protein structure of the photosensor VVD in the light-induced dimeric state (PDB code: 3RH8, https://doi.org/10.2210/pdb3RH8/pdb[2]), with (A) amino acid residue T69 in the Ncap of one monomer highlighted as red spheres and in (E) with amino acid residue N100 at the surface of the PAS core in one monomer highlighted as red spheres. nMagHigh1 is shown in yellow and its Ncap in orange. pMag is colored sky blue and its Ncap in darker blue. mCherry expression levels obtained at either high-intensity light induction at 3.78 W m^{-2} shown in light blue bars, low-intensity light induction at 0.95 W m^{-2} shown in dark blue bars as well as grown in the dark shown in black, each at steady state after 5h growth in the respective condition for (B) selected T69 variants and (F) selected N100 variants. The light sensitivity of the same variants at low light induction is plotted relative to the WT for (C) T69 and (G) N100 variants. Relative fold-change of dark-to-light expression level compared to the WT for (D) T69 and (H) N100 variants. (A, E) show

normalized expression levels of individual samples relative to the mean mCherry expression value of the WT which consist of three biological triplicates. Diagrams show mean mCherry expression values and standard deviation (mean values +/- SD) of three biological replicates measured after 5h incubation time for (B, F). Relative light sensitivity is calculated from these values by normalizing the mean mCherry expression and standard deviation (mean values +/- SD) of variants to the mean mCherry expression of the WT (C, G). Relative fold changes were calculated as the ratio of light-induced to dark controls of the individual samples and the standard error of the ratio, which was again normalized to the fold change of the WT under the respective conditions (D, H).

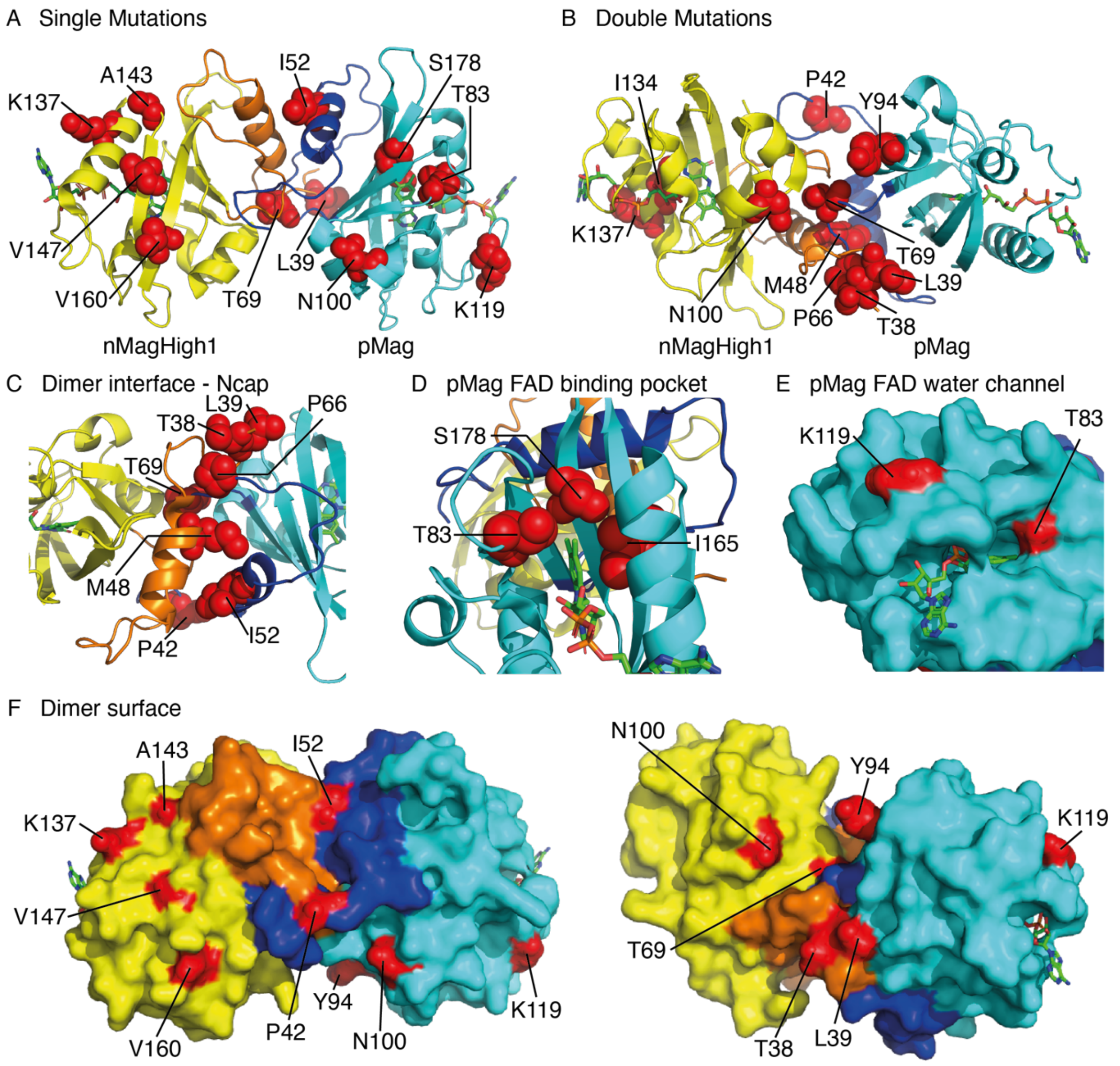

Supplementary Figure 29: Positions of identified mutations in the light-induced VVD homodimer PDB code: 3RH8 [https://doi.org/10.2210/pdb3RH8/pdb]. All shown protein structures depict a light-induced VVD homodimer which serve as representation of Magnets that differ in 2 positions for pMag (I52R M55R) and nMagHigh1 (I52D M55G M135I M165I). nMagHigh1 is exemplarily shown in yellow and its Ncap in orange. pMag is colored cyan and its Ncap in blue. All shown amino acids are therefore the positions in the VVD photoregulator. The positions of identified mutations are shown in one letter codes next to the residues marked in red. Residue positions from identified (A) single mutations and found in (B) double mutants are highlighted as red spheres. (C) Mutations in the VVD Ncap: T38A, L39P/R, and P42T are located in the N-terminal "latch" (amino acid residues 37 – 44) that wraps around the domain, M48V and R52L are located in the interface of the dimer within the subsequent Aα helix, and P66S and T69M are located in the hinge region of the dimer interface. (C) Detail of flavin binding pocket with, T83, S178 and I165, for which T83M, M165T, and S178Y variants were identified. Residues are shown in red spheres. (E) Surface detail of the entrance of the water channel harboring the flavin chromophore with T83 residue and K119 residue located in the FAD loop shown in light blue. Both residues are shown in red. (F) Surface of the dimer protein with residues of identified variants shown in red.

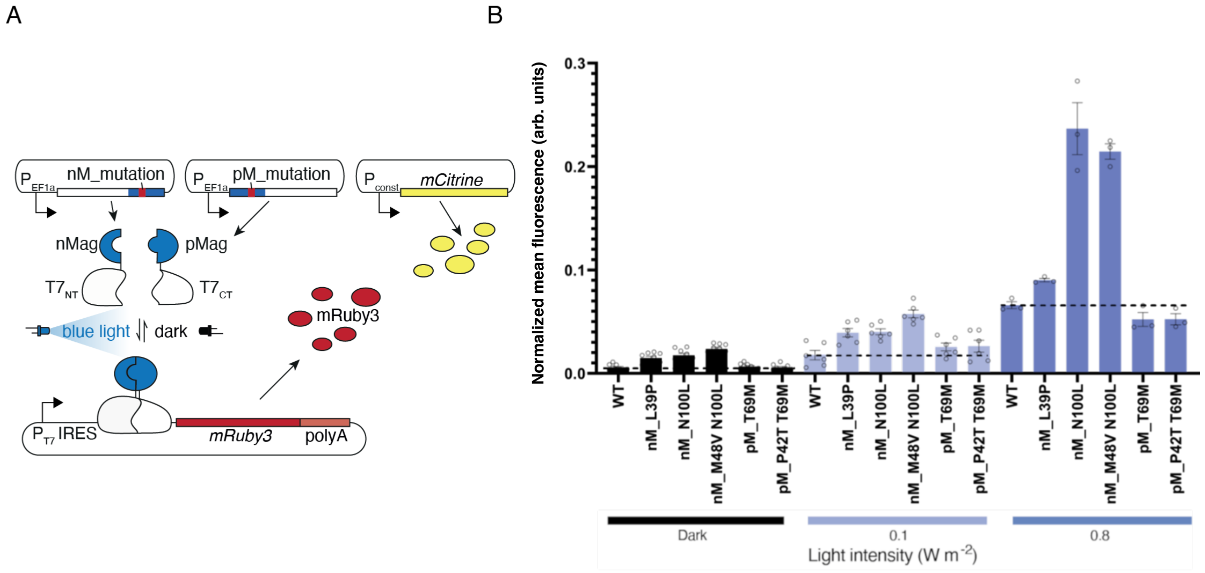

Supplementary Figure 30: Characterization of selected Magnet variants in mammalian cells. (A) Plasmids used for transfection of HEK293T cells. The mOptoT7 fragments are expressed from EF1a promoters and a separate plasmid for each of the components. The mutations were either located in nMagHigh1 (nM_mutation) or pMag (pM_mutation). mOptoT7 drives the transcription of mRuby3. mCitrine is used to normalize the mRuby3 expression to the transfection efficiency. (B) Normalized mean fluorescence of mRuby3 expression in the dark and after light induction of the wild type mOptoT7 (WT) and selected identified variants. Induction was performed at a sub-saturating light-intensity (0.1 W m^{-2}) and saturating light-intensity (0.8 W m^{-2}). Shown are normalized fluorescence values as mean with standard deviation (mean values +/- SD) as well as individual points of at least three biological replicates (n=3).

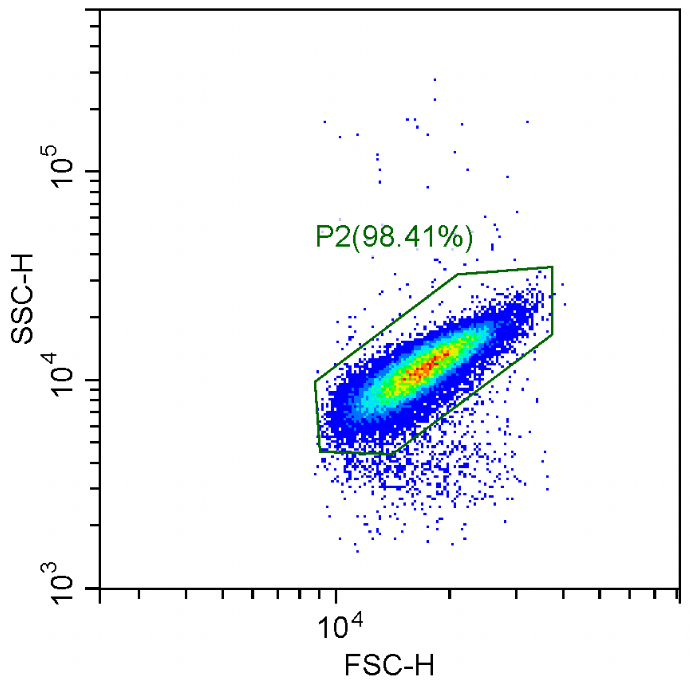

Supplementary Figure 31: Flow cytometry gating strategies. Gate used for the analysis of flow cytometric data of all samples. The SSC-H and FSC-H hexagon gate was drawn by eye on the AB360 control in the CytExpert v.2.1.0.92 and kept constant for all experiments using the same cell type. Shown is a screenshot of gates taken directly from the CytExpert software.

Supplementary Table 1: Identified variant mutations in the respective Magnet domain, summarized as discovered single or double mutations.

nMagHigh1		pMag	
single	double	single	double
L39P	T38A, L39R	R52L	P42T, T69M
K137E	M48V, N100L	T69M	P66S, Y94N
A143S	I135V, K137R	T83M	
V147M		N100K	
V160M		N100L	
		N100S	
		K119N	
		M165T	
		S178Y	

Supplementary Table 2: Parameters of dose-response curve fits of Opto-T7RNAP*(563) (Wild Type; WT) and nMagHigh1 variants, grown for 5h at 37°C and measured through flow cytometry. Fitting was performed as described in the Methods section.

nMagHigh1 variants at 37°C	WT	L39P	K137E	A143S	V147M	V160M	T38A L39R	M48V N100L	I135V K137R
Best-fit values									
Basal expression (*b*) arb. units	623	936	831	569	552	923	520	1845	547
Maximal expression (*t*) arb. units	28360	42942	36409	27030	26910	41987	21709	75667	31394
Change (*t*/*b*)	46	46	44	48	49	46	42	41	57
Half-maximal intensity (*I50*) W m^{-2}	0.67	0.53	0.66	0.96	1.08	0.56	0.45	0.33	1.06
Hillslope (*n*)	1.7	1.6	1.5	1.7	1.7	1.5	1.7	2.4	1.6
Lower 95% conf. limit (profile likelihood)									
Basal expression (*b*) arb. units	-1816	-2892	-1329	-1262	-1454	-1887	-1267	-3141	-1454
Maximal expression (*t*) arb. units	26147	39599	34165	24979	24504	39324	20311	72507	28789
Half-maximal intensity (*I50*) W m^{-2}	0.53	0.40	0.55	0.79	0.88	0.46	0.34	0.22	0.87
Hillslope (*n*)	1.1	0.9	1.1	1.2	1.2	1.0	1.0	1.3	1.2
Upper 95% conf. limit (profile likelihood)									
Basal expression (*b*) arb. units	3065	4768	2994	2401	2554	3735	2309	6831	2547
Maximal expression (*t*) arb. units	32079	49067	39885	30386	31328	46215	23882	79543	36033
Half-maximal intensity (*I50*) W m^{-2}	0.86	0.70	0.80	1.21	1.45	0.68	0.56	0.42	1.39
Hillslope (*n*)	2.4	2.4	2.0	2.3	2.4	2.1	2.7	6.7	2.2
Goodness of Fit									
Degrees of Freedom	32	32	32	32	32	32	32	32	32
R squared	0.926	0.919	0.962	0.954	0.944	0.952	0.931	0.956	0.957
Comparison to WT (in %)									
change in *b*	100	150	133	91	89	148	84	296	88
change in *t*	100	151	128	95	95	148	77	267	111
change *t*/*b*	100	101	96	104	107	100	92	90	126
change in *I50*	100	80	98	143	162	83	68	50	158

Supplementary Table 3: Parameters of dose-response curve fits of Opto-T7RNAP*(563) (Wild Type; WT) and nMagHigh1 variants, grown for 8h at 37°C and measured through spectrophotometry. Fitting was performed as described in the Methods section.

nMagHigh1 variants at 37°C	WT	L39P	K137E	A143S	V147M	V160M	T38A L39R	M48V N100L	I135V K137R
Best-fit values									
Basal expression (*b*) arb. units	73	82	81	77	75	80	74	103	81
Maximal expression (*t*) arb. units	745	1107	808	619	639	990	528	1552	610
Change (*t*/*b*)	10	14	10	8	9	12	7	15	8
Half-maximal intensity (*I50*) W m^{-2}	0.59	0.41	0.61	0.95	0.99	0.52	0.42	0.31	1.05
Hillslope (*n*)	1.8	1.3	1.8	1.8	1.8	1.7	2.0	2.1	2.0
Lower 95% conf. limit (profile likelihood)									
Basal expression (*b*) arb. units	-14.22	-37.83	-36.35	4.674	22.41	-20.12	26.03	-112	30.73
Maximal expression (*t*) arb. units	678.1	992.7	715.9	549.3	585.6	908.1	494.8	1418	560.7
Half-maximal intensity (*I50*) W m^{-2}	0.43	0.22	0.39	0.68	0.78	0.37	0.27	0.02	0.84
Hillslope (*n*)	0.9	0.3	0.6	0.9	1.2	0.8	1.0	0.1	1.3
Upper 95% conf. limit (profile likelihood)									
Basal expression (*b*) arb. units	159.9	201.5	198.4	148.1	126.8	180.9	122.5	318.2	130.7
Maximal expression (*t*) arb. units	873.3	2625	1142	798.7	734.2	1168	577.9	???	692.8
Half-maximal intensity (*I50*) W m^{-2}	0.83	21.03	1.38	1.67	1.32	0.70	0.53	???	1.36
Hillslope (*n*)	3.0	2.8	3.6	3.2	2.7	2.9	3.9	???	3.0
Goodness of Fit									
Degrees of Freedom	47	46	50	50	50	50	49	50	49
R squared	0.802	0.812	0.702	0.782	0.882	0.830	0.847	0.737	0.885
Comparison to WT (in %)									
change in *b*	100	113	111	105	103	110	102	142	111
change in *t*	100	149	109	83	86	133	71	208	82
change *t*/*b*	100	132	98	79	84	120	69	147	74
change in *I50*	100	70	102	160	167	87	70	52	178

Supplementary Table 4: Parameters of dose-response curve fits of Opto-T7RNAP*(563) (Wild Type; WT) and pMag variants, grown for 5h at 37°C and measured through flow cytometry. Fitting was performed as described in the Methods section.

pMag variants at 37°C	WT	R52L	T69M	T83M	N100K	N100L	N100S	K119N	M165T	S178Y	P42T T69M	P66S Y94N
Best-fit values												
Basal expression (*b*) arb. units	622.8	1497	1601	1444	902.5	2253	944.9	753.3	2042	1361	2629	1431
Maximal expression (*t*) arb. units	28360	78130	92569	76702	53898	153134	47240	29672	61087	71816	146536	56961
Change (*t/b*)	46	52	58	53	60	68	50	39	30	53	56	40
Half-maximal intensity (*I50*) W m^{-2}	0.67	0.53	0.34	0.32	0.48	0.39	0.59	0.52	0.34	0.43	0.35	0.55
Hillslope (*n*)	1.7	1.7	2.0	1.8	1.7	1.6	1.5	1.8	3.5	1.8	1.9	1.9
Lower 95% conf. limit (profile likelihood)												
Basal expression (*b*) arb. units	-1816	-5194	-6660	-3208	-2829	-4621	-1795	-1672	-3684	-3558	-5004	-2954
Maximal expression (*t*) arb. units	26147	72687	87059	73412	50775	147061	44369	27772	57938	68056	141112	53515
Half-maximal intensity (*I50*) W m^{-2}	0.53	0.41	0.19	0.22	0.38	0.33	0.50	0.42	0.14	0.34	0.28	0.45
Hillslope (*n*)	1.1	1.1	0.9	1.1	1.1	1.1	1.1	1.2	1.2	1.2	1.3	1.3
Upper 95% conf. limit (profile likelihood)												
Basal expression (*b*) arb. units	3065	8197	9864	6097	4636	9127	3686	3181	7767	6281	10263	5821
Maximal expression (*t*) arb. units	32079	86549	100844	81171	58519	161570	51891	32401	64583	76997	153253	61628
Half-maximal intensity (*I50*) W m^{-2}	0.86	0.66	0.43	0.39	0.58	0.45	0.72	0.64	???	0.51	0.41	0.67
Hillslope (*n*)	2.4	2.6	4.7	3.1	2.3	2.1	2.0	2.7	???	2.7	2.9	2.6
Goodness of Fit												
Degrees of Freedom	32	32	32	32	32	32	32	32	32	32	32	32
R squared	0.926	0.927	0.922	0.962	0.950	0.978	0.963	0.933	0.916	0.952	0.972	0.941
Comparison to WT (in %)												
change in *b*	100	240	257	232	145	362	152	121	328	219	422	230
change in *t*	100	275	326	270	190	540	167	105	215	253	517	201
change *t/b*	100	115	127	117	131	149	110	87	66	116	122	87
change in *I50*	100	79	50	47	71	59	89	78	50	64	52	83

Supplementary Table 5: Parameters of dose-response curve fits of Opto-T7RNAP*(563) (Wild Type; WT) and pMag variants, grown for 8h at 37°C and measured through spectrophotometry. Fitting was performed as described in the Methods section.

pMag variants at 37°C	WT	R52L	T69M	T83M	N100K	N100L	N100S	K119N	M165T	S178Y	P42T T69M	P66S Y94N
Best-fit values												
Basal expression (*b*) arb. units	73	90	91	88	83	103	78	74	103	86	116	92
Maximal expression (*t*) arb. units	745	1697	1916	1616	1110	2055	943.3	676.3	1335	1638	3219	1187
Change (*t*/*b*)	10	19	21	18	13	20	12	9	13	19	28	13
Half-maximal intensity (*I50*) W m^{-2}	0.59	0.47	0.35	0.33	0.52	0.42	0.52	0.46	0.37	0.34	0.32	0.49
Hillslope (*n*)	1.8	2.1	2.5	3.0	1.6	1.8	2.0	2.2	6.2	1.8	2.3	2.1
Lower 95% conf. limit (profile likelihood)												
Basal expression (*b*) arb. units	-14.22	-131.8	-133.4	-110.4	-91.09	-490.3	6.253	15.08	-51.58	-66.71	-309.6	-77.38
Maximal expression (*t*) arb. units	678.1	1547	1780	1502	970.5	1647	889.8	637.3	1256	1528	2956	1074
Half-maximal intensity (*I50*) W m^{-2}	0.43	0.30	0.13	0.02	0.27	0.00	0.42	0.35	???	0.17	0.04	0.30
Hillslope (*n*)	0.9	0.9	0.7	0.3	0.3	???	1.3	1.3	0.4	0.7	0.2	0.9
Upper 95% conf. limit (profile likelihood)												
Basal expression (*b*) arb. units	159.9	311.9	315.4	287.2	256.5	695.4	149.8	133.3	258.2	239.4	541.6	261.5
Maximal expression (*t*) arb. units	873.3	1938	2168	2122	6311	???	1013	723.3	1434	1869	6606	1382
Half-maximal intensity (*I50*) W m^{-2}	0.83	0.63	0.45	???	1815.00	???	0.63	0.56	???	0.45	6.19	0.69
Hillslope (*n*)	3.0	4.8	???	???	4.3	???	2.8	3.8	???	4.2	???	4.5
Goodness of Fit												
Degrees of Freedom	47	49	50	49	50	49	50	50	50	48	50	50
R squared	0.802	0.769	0.806	0.791	0.673	0.395	0.901	0.868	0.803	0.865	0.767	0.725
Comparison to WT (in %)												
change in *b*	100	124	125	122	114	141	107	102	142	119	160	126
change in *t*	100	228	257	217	149	276	127	91	179	220	432	159
change *t*/*b*	100	184	206	179	131	195	118	89	126	185	271	126
change in *I50*	100	79	60	55	88	71	88	78	63	58	55	83

Supplementary Table 6: Parameters of dose-response curve fits of Opto-T7RNAP*(563) (Wild Type; WT) and nMagHigh1 variants, grown for 5h at 30°C and measured through flow cytometry. Fitting was performed as described in the Methods section.

nMagHigh1 variants at 30°C	WT	L39P	K137E	A143S	V147M	V160M	T38A L39R	M48V N100L	I135V K137R
Best-fit values									
Basal expression (*b*) arb. units	12539	14867	11310	8456	5892	15556	9956	33974	5147
Maximal expression (*t*) arb. units	128601	141254	133081	117004	125381	129201	98245	140683	136077
Change (*t*/*b*)	10	10	12	14	21	8	10	4	26
Half-maximal intensity (*I50*) W m^{-2}	0.17	0.15	0.16	0.17	0.21	0.14	0.13	0.09	0.20
Hillslope (*n*)	1.3	1.2	1.4	1.7	1.6	1.5	1.4	1.6	1.3
Lower 95% conf. limit (profile likelihood)									
Basal expression (*b*) arb. units	9720	9218	6722	3627	456.1	9491	6771	28669	-75.39
Maximal expression (*t*) arb. units	122353	129175	124277	109959	115637	119450	92783	134108	124188
Half-maximal intensity (*I50*) W m^{-2}	0.16	0.12	0.14	0.15	0.18	0.12	0.11	0.08	0.17
Hillslope (*n*)	1.1	0.9	1.1	1.4	1.3	1.0	1.1	1.1	1.0
Upper 95% conf. limit (profile likelihood)									
Basal expression (*b*) arb. units	15357	20514	15893	13280	11296	21618	13140	39279	10362
Maximal expression (*t*) arb. units	136930	164289	146345	126535	139879	145738	106163	151192	156207
Half-maximal intensity (*I50*) W m^{-2}	0.20	0.22	0.20	0.20	0.26	0.18	0.15	0.11	0.27
Hillslope (*n*)	1.5	1.6	1.7	2.1	2.0	2.0	1.8	2.3	1.7
Goodness of Fit									
Degrees of Freedom	14	14	14	14	14	14	14	14	14
R squared	0.997	0.989	0.993	0.991	0.990	0.986	0.994	0.989	0.991
Comparison to WT (in %)									
change in *b*	100	119	90	67	47	124	79	271	41
change in *t*	100	110	103	91	97	100	76	109	106
change *t*/*b*	100	93	115	135	207	81	96	40	258
change in *I50*	100	89	93	98	121	82	75	52	116

Supplementary Table 7: Parameters of dose-response curve fits of Opto-T7RNAP*(563) (Wild Type; WT) and nMagHigh1 variants, grown for 13h at 30°C and measured through spectrophotometry. Fitting was performed as described in the Methods section.

nMagHigh1 variants at 30°C	WT	L39P	K137E	A143S	V147M	V160M	T38A L39R	M48V N100L	I135V K137R
Best-fit values									
Basal expression (*b*) arb. units	944	1939	1275	525	442	1486	1033	3591	477
Maximal expression (*t*) arb. units	14995	17252	16329	13107	14849	15913	10982	17311	14767
Change (*t*/*b*)	16	9	13	25	34	11	11	5	31
Half-maximal intensity (*I50*) W m^{-2}	0.15	0.12	0.14	0.18	0.22	0.12	0.12	0.08	0.19
Hillslope (*n*)	1.5	1.6	1.6	1.7	1.7	1.7	1.6	1.2	1.6
Lower 95% conf. limit (profile likelihood)									
Basal expression (*b*) arb. units	277	1370	704.4	32.33	-456.5	941	488.6	2543	-112.5
Maximal expression (*t*) arb. units	13863	16476	15492	12358	13365	15128	10250	15919	13805
Half-maximal intensity (*I50*) W m^{-2}	0.13	0.10	0.13	0.16	0.18	0.11	0.10	0.05	0.17
Hillslope (*n*)	1.1	1.3	1.3	1.4	1.3	1.3	1.1	0.5	1.3
Upper 95% conf. limit (profile likelihood)									
Basal expression (*b*) arb. units	1611	2508	1846	1016	1335	2031	1578	4639	1066
Maximal expression (*t*) arb. units	16756	18271	17442	14094	17268	16966	12156	22409	16105
Half-maximal intensity (*I50*) W m^{-2}	0.19	0.13	0.17	0.21	0.28	0.14	0.14	0.15	0.22
Hillslope (*n*)	1.9	2.0	2.0	2.0	2.3	2.1	2.2	2.3	2.0
Goodness of Fit									
Degrees of Freedom	40	41	49	50	46	44	46	47	49
R squared	0.965	0.980	0.974	0.972	0.935	0.977	0.951	0.907	0.968
Comparison to WT (in %)									
change in *b*	100	205	135	56	47	157	109	380	51
change in *t*	100	115	109	87	99	106	73	115	98
change *t*/*b*	100	56	81	157	212	67	67	30	195
change in *I50*	100	75	94	117	143	78	77	51	124

Supplementary Table 8: Parameters of dose-response curve fits of Opto-T7RNAP*(563) (Wild Type; WT) and pMag variants, grown for 5h at 30°C and measured through flow cytometry. Fitting was performed as described in the Methods section.

pMag variants at 30°C	WT	R52L	T69M	T83M	N100K	N100L	N100S	K119N	M165T	S178Y	P42T T69M	P66S Y94N
Best-fit values												
Basal expression (*b*) arb. units	12539	41431	32604	30733	23084	31754	14001	11358	30641	22991	58643	21119
Maximal expression (*t*) arb. units	128601	264567	170188	161146	112736	212277	145632	125617	147397	174306	265693	212713
Change (*t*/*b*)	10	6	5	5	5	7	10	11	5	8	5	10
Half-maximal intensity (*I50*) W m^{-2}	0.17	0.11	0.10	0.09	0.09	0.13	0.16	0.15	0.11	0.13	0.10	0.13
Hillslope (*n*)	1.3	1.5	1.5	1.6	2.5	1.3	1.2	1.5	1.5	1.2	1.4	1.5
Lower 95% conf. limit (profile likelihood)												
Basal expression (*b*) arb. units	9720	33344	24785	26645	18332	24292	8821	6529	23695	18660	51504	12309
Maximal expression (*t*) arb. units	122353	252321	159804	155571	108973	197244	134022	117411	138046	164868	254858	199673
Half-maximal intensity (*I50*) W m^{-2}	0.16	0.10	0.08	0.08	0.08	0.11	0.13	0.13	0.09	0.12	0.09	0.11
Hillslope (*n*)	1.1	1.1	0.9	1.2	1.8	0.9	0.9	1.1	1.0	1.0	1.1	1.2
Upper 95% conf. limit (profile likelihood)												
Basal expression (*b*) arb. units	15357	49517	40422	34820	27836	39213	19178	16182	37587	27321	65782	29925
Maximal expression (*t*) arb. units	136930	281954	189266	168629	117505	239148	166200	137897	164315	188336	281420	232156
Half-maximal intensity (*I50*) W m^{-2}	0.20	0.13	0.12	0.10	0.10	0.17	0.21	0.18	0.13	0.16	0.11	0.15
Hillslope (*n*)	1.5	1.9	2.3	2.0	4.7	1.7	1.6	1.9	2.2	1.5	1.8	2.0
Goodness of Fit												
Degrees of Freedom	14	14	14	14	14	14	14	14	14	14	14	14
R squared	0.997	0.994	0.986	0.996	0.989	0.991	0.992	0.991	0.984	0.996	0.995	0.990
Comparison to WT (in %)												
change in *b*	100	330	260	245	184	253	112	91	244	183	468	168
change in *t*	100	206	132	125	88	165	113	98	115	136	207	165
change *t*/*b*	100	62	51	51	48	65	101	108	47	74	44	98
change in *I50*	100	63	55	54	54	75	90	87	61	77	56	74

Supplementary Table 9: Parameters of dose-response curve fits of Opto-T7RNAP*(563) (Wild Type; WT) and pMag variants, grown for 15h for N100L and 13h for all other variants at 30°C and measured through spectrophotometry. Fitting was performed as described in the Methods section.

pMag variants at 30°C	WT	R52L	T69M	T83M	N100K	N100L	N100S	K119N	M165T	S178Y	P42T T69M	P66S Y94N
Best-fit values												
Basal expression (*b*) arb. units	944	4055	3075	2797	2049	3412	1338	1023	2821	2298	6849	2596
Maximal expression (*t*) arb. units	14995	29401	22549	21261	14750	20904	16109	14050	17345	20888	28763	25232
Change (*t/b*)	16	7	7	8	7	6	12	14	6	9	4	10
Half-maximal intensity (*I50*) W m^{-2}	0.15	0.08	0.08	0.08	0.09	0.08	0.13	0.15	0.11	0.11	0.04	0.12
Hillslope (*n*)	1.5	2.2	1.9	1.6	1.9	2.8	1.7	1.6	1.5	1.5	1.2	1.9
Lower 95% conf. limit (profile likelihood)												
Basal expression (*b*) arb. units	277	2434	2279	2082	669.5	1628	863.7	559	2176	1657	5164	1800
Maximal expression (*t*) arb. units	13863	28111	21756	20447	13511	19754	15470	13376	16433	19981	26814	24357
Half-maximal intensity (*I50*) W m^{-2}	0.13	0.06	0.07	0.07	0.06	0.06	0.12	0.13	0.09	0.09	0.01	0.11
Hillslope (*n*)	1.1	1.2	1.3	1.2	0.8	1.2	1.4	1.4	1.1	1.2	0.1	1.6
Upper 95% conf. limit (profile likelihood)												
Basal expression (*b*) arb. units	1611	5677	3870	3511	3428	5196	1812	1486	3465	2939	8535	3392
Maximal expression (*t*) arb. units	16756	31442	23617	22384	17719	22931	16900	14924	18754	22122	???	26284
Half-maximal intensity (*I50*) W m^{-2}	0.19	???	0.09	0.09	0.13	0.10	0.15	0.16	0.13	0.12	???	0.13
Hillslope (*n*)	1.9	???	2.6	2.2	???	???	2.0	2.0	2.0	1.9	???	2.3
Goodness of Fit												
Degrees of Freedom	40	49	50	50	49	50	50	49	49	50	50	50
R squared	0.965	0.939	0.973	0.975	0.838	0.860	0.982	0.978	0.965	0.979	0.906	0.980
Comparison to WT (in %)												
change in *b*	100	429	326	296	217	361	142	108	299	243	725	275
change in *t*	100	196	150	142	98	139	107	94	116	139	192	168
change *t/b*	100	46	46	48	45	39	76	86	39	57	26	61
change in *I50*	100	49	53	54	61	54	87	94	69	69	26	75

Supplementary Table 10: Fluorescence (mCherry) expression of WT and nMagHigh1 variants at 40°C. Shown is the mean fluorescence of three biological replicates (n=3) and percentage of fluorescence from the different variants in comparison with the WT

nMagHigh1 variants at 40°C	WT	L39P		K137E		A143S		V147M		V160M		T38A L39R		M48V N100L		I135V K137R	
light intensity (W m⁻²)	Fluo AVG (arb. units)	Fluo AVG (arb. units)	%	Fluo AVG (arb. units)	%	Fluo AVG (arb. units)	%	Fluo AVG (arb. units)	%	Fluo AVG (arb. units)	%	Fluo AVG (arb. units)	%	Fluo AVG (arb. units)	%	Fluo AVG (arb. units)	%
0	82	96	118	92	113	85	105	80	98	90	110	84	103	135	165	83	102
0.48	128	189	148	173	135	103	81	93	72	201	157	128	100	438	342	102	80
0.96	176	246	140	226	128	134	76	123	70	260	147	157	89	604	343	135	77
1.92	223	337	151	303	136	172	77	152	68	368	165	205	92	824	369	175	78
3.85	252	364	144	317	126	194	77	176	70	396	157	221	88	878	348	202	80
5.77	305	474	155	389	127	250	82	240	79	517	169	270	89	1256	412	260	85
maximal fold change	3.7	4.9		4.2		2.9		3.0		5.8		3.2		9.3		3.1	
difference of fold change to WT (%)	100	132		113		78		80		154		86		249		83	

Supplementary Table 11: Fluorescence (mCherry) expression of WT and pMag variants at 40°C. Shown is the mean fluorescence of three biological replicates (n=3) and percentage of fluorescence from the different variants in comparison with the WT

pMag variants at 40°C	WT	R52L		T69M		T83M		N100K		N100L		N100S		K119N		M165T	
light intensity (W m⁻²)	Fluo AVG (arb. units)	Fluo AVG (arb. units)	%	Fluo AVG (arb. units)	%	Fluo AVG (arb. units)	%	Fluo AVG (arb. units)	%	Fluo AVG (arb. units)	%	Fluo AVG (arb. units)	%	Fluo AVG (arb. units)	%	Fluo AVG (arb. units)	%
0	82	124	153	134	164	118	144	105	129	129	159	98	120	96	118	143	175
0.48	128	232	181	426	332	365	285	275	215	856	668	172	135	141	110	378	295
0.96	176	333	189	593	337	491	279	343	195	944	536	229	130	189	107	498	283
1.92	223	436	195	830	372	699	313	485	218	1442	647	306	137	219	98	612	274
3.85	252	520	206	944	374	746	296	550	218	1634	648	345	137	250	99	643	255
5.77	305	655	215	1243	407	1065	349	726	238	2386	782	444	145	306	100	777	255
maximal fold change	3.7	5.3		9.3		9.1		6.9		18.5		4.5		3.2		5.4	
difference of fold change to WT (%)	100	141		248		242		184		493		121		85		145	

Supplementary Table 12: Fluorescence (mCherry) expression of WT and pMag variants at 40°C. Shown is the mean fluorescence of three biological replicates (n=3) and percentage of fluorescence from the different variants in comparison with the WT.

pMag variants at 40°C	WT	S178Y		P42T T69M	
light intensity (W m^{-2})	Fluo AVG (arb. units)	Fluo AVG (arb. units)	%	Fluo AVG (arb. units)	%
0.0	82	115	141	213	261
0.5	128	326	254	619	484
1.0	176	453	257	937	532
1.9	223	646	290	1389	623
3.9	252	777	308	1720	682
5.8	305	1043	342	2381	781
maximal fold change	3.7	9.1		11.2	
difference of fold change to WT (%)	100	243		299	

Supplementary Table 13: Parameters of growth curve fits of negative control (NC), Opto-T7RNAP*(563) (Wild Type; WT) and Opto-T7RNAP*(563) variant pMag P42T T69M, grown at 30°C and absorbance at 600 nm measured through spectrophotometry and corresponding to the data shown in Supplementary Figure 21. Fitting was performed as described in the Methods section.

	NC						WT						pMag P42T T69M					
	Dark	0.48 Wm-2	0.96 Wm-2	1.92 Wm-2	3.85 Wm-2	5.77 Wm-2	Dark	0.48 Wm-2	0.96 Wm-2	1.92 Wm-2	3.85 Wm-2	5.77 Wm-2	Dark	0.48 Wm-2	0.96 Wm-2	1.92 Wm-2	3.85 Wm-2	5.77 Wm-2
Best-fit values																		
Basal absorbance (b) arb. units	0.07602	0.07623	0.07566	0.07505	0.07521	0.07599	0.07428	0.07405	0.07348	0.07293	0.07323	0.07226	0.0757	0.0702	0.07012	0.07097	0.07149	0.07117
Maximal absorbance (t) arb. units	0.6902	0.6882	0.6883	0.6889	0.6908	0.6782	0.6967	0.7107	0.7096	0.7172	0.713	0.7282	0.6977	0.7395	0.7397	0.7411	0.7217	0.7435
T50 (h)	11.52	11	10.6	10.6	10.39	10.43	11.91	11.7	11.46	11.77	11.39	11.7	11.8	13.06	13.52	13.71	13.5	13.91
Hill slope (n)	0.3096	0.3164	0.3165	0.3191	0.3176	0.329	0.302	0.2969	0.2871	0.2704	0.2727	0.2631	0.3013	0.216	0.2008	0.2032	0.2022	0.1941
Absorbance increase (t-b) arb. units	0.6142	0.612	0.6126	0.6138	0.6156	0.6022	0.6225	0.6366	0.6361	0.6443	0.6398	0.6559	0.622	0.6693	0.6696	0.6701	0.6502	0.6723
95% CI (profile likelihood)																		
Basal absorbance (b) arb. units	0.06973 to 0.08222	0.07034 to 0.08205	0.06732 to 0.08384	0.06630 to 0.08364	0.06646 to 0.08380	0.06582 to 0.08594	0.06722 to 0.08125	0.06613 to 0.08185	0.06511 to 0.08172	0.06383 to 0.08185	0.06448 to 0.08182	0.06390 to 0.08046	0.06814 to 0.08314	0.06335 to 0.07691	0.06270 to 0.07735	0.06322 to 0.07852	0.06316 to 0.07958	0.06306 to 0.07904
Maximal absorbance (t) arb. units	0.6840 to 0.6965	0.6829 to 0.6937	0.6811 to 0.6956	0.6813 to 0.6965	0.6834 to 0.6982	0.6697 to 0.6869	0.6889 to 0.7047	0.7021 to 0.7194	0.7008 to 0.7186	0.7072 to 0.7275	0.7037 to 0.7226	0.7190 to 0.7376	0.6900 to 0.7055	0.7304 to 0.7488	0.7289 to 0.7512	0.7294 to 0.7535	0.7097 to 0.7344	0.7306 to 0.7574
T50 (h)	11.43 to 11.62	10.91 to 11.08	10.48 to 10.71	10.47 to 10.72	10.27 to 10.51	10.29 to 10.57	11.79 to 12.02	11.57 to 11.83	11.32 to 11.59	11.62 to 11.93	11.24 to 11.53	11.56 to 11.84	11.68 to 11.91	12.93 to 13.18	13.38 to 13.67	13.55 to 13.86	13.34 to 13.67	13.75 to 14.08
Hill slope (n)	0.2921 to 0.3286	0.3000 to 0.3341	0.2942 to 0.3412	0.2954 to 0.3454	0.2944 to 0.3433	0.3006 to 0.3612	0.2819 to 0.3241	0.2756 to 0.3204	0.2660 to 0.3103	0.2493 to 0.2937	0.2523 to 0.2952	0.2448 to 0.2831	0.2812 to 0.3231	0.2043 to 0.2284	0.1890 to 0.2134	0.1904 to 0.2168	0.1885 to 0.2168	0.1813 to 0.2078
Goodness of Fit																		
Degrees of Freedom	212	212	212	212	212	212	196	197	172	197	149	172	212	212	212	212	212	212
R squared	0.9917	0.9932	0.9872	0.986	0.9865	0.9806	0.9894	0.9876	0.9884	0.9845	0.9892	0.9892	0.9881	0.9915	0.9898	0.9884	0.9865	0.9875

Supplementary Table 14: Parameters of growth curve fits of Opto-T7RNAP*(563) variants pMag R52L, pMag N100L and pMag P66S Y94N, grown at 30°C and absorbance at 600 nm measured through spectrophotometry and corresponding to the data shown in Supplementary Figure 21. Fitting was performed as described in the Methods section.

	pMag R52L						pMag N100L						pMag P66S Y94N					
	Dark	0.48 Wm-2	0.96 Wm-2	1.92 Wm-2	3.85 Wm-2	5.77 Wm-2	Dark	0.48 Wm-2	0.96 Wm-2	1.92 Wm-2	3.85 Wm-2	5.77 Wm-2	Dark	0.48 Wm-2	0.96 Wm-2	1.92 Wm-2	3.85 Wm-2	5.77 Wm-2
Best-fit values																		
Basal absorbance (b) arb. units	0.07553	0.07131	0.07046	0.06932	0.06892	0.06839	0.07656	0.07619	0.07653	0.07449	0.07289	0.07206	0.07633	0.07498	0.07175	0.07407	0.07218	0.07083
Maximal absorbance (t) arb. units	0.6833	0.7137	0.7147	0.7309	0.7257	0.7356	0.6448	0.6624	0.6699	0.6796	0.6838	0.679	0.6847	0.7101	0.7242	0.7099	0.7261	0.734
T50 (h)	11.57	12.24	13.07	13.46	13.38	13.78	12.81	13.34	13.73	13.81	13.76	14	11.54	11.8	12.29	12.44	12.45	12.9
Hill slope (n)	0.3127	0.2454	0.2062	0.1968	0.1904	0.1858	0.2736	0.2429	0.2214	0.2146	0.2036	0.2012	0.3123	0.2766	0.2371	0.239	0.228	0.2174
Absorbance increase (t-b) arb. units	0.6078	0.6424	0.6443	0.6616	0.6567	0.6672	0.5682	0.5862	0.5933	0.6051	0.6109	0.607	0.6084	0.6351	0.6524	0.6359	0.654	0.6631
95% CI (profile likelihood)																		
Basal absorbance (b) arb. units	0.06684 to 0.08406	0.06318 to 0.07927	0.05939 to 0.08109	0.05982 to 0.07848	0.05890 to 0.07853	0.05841 to 0.07797	0.06896 to 0.08400	0.06838 to 0.08382	0.06788 to 0.08493	0.06392 to 0.08467	0.06099 to 0.08424	0.05822 to 0.08517	0.06951 to 0.08306	0.06770 to 0.08214	0.06434 to 0.07900	0.06578 to 0.08216	0.06369 to 0.08044	0.06268 to 0.07877
Maximal absorbance (t) arb. units	0.6748 to 0.6920	0.7046 to 0.7230	0.7004 to 0.7301	0.7173 to 0.7455	0.7114 to 0.7411	0.7200 to 0.7528	0.6358 to 0.6540	0.6521 to 0.6732	0.6573 to 0.6832	0.6640 to 0.6965	0.6663 to 0.7032	0.6575 to 0.7035	0.6780 to 0.6915	0.7027 to 0.7177	0.7158 to 0.7327	0.7005 to 0.7197	0.7164 to 0.7362	0.7236 to 0.7448
T50 (h)	11.44 to 11.70	12.10 to 12.37	12.86 to 13.28	13.27 to 13.65	13.18 to 13.59	13.57 to 13.99	12.67 to 12.95	13.19 to 13.50	13.55 to 13.92	13.59 to 14.04	13.51 to 14.02	13.70 to 14.33	11.44 to 11.65	11.69 to 11.92	12.17 to 12.42	12.30 to 12.59	12.30 to 12.59	12.75 to 13.05
Hill slope (n)	0.2884 to 0.3397	0.2291 to 0.2630	0.1885 to 0.2257	0.1822 to 0.2126	0.1756 to 0.2063	0.1711 to 0.2017	0.2531 to 0.2963	0.2247 to 0.2628	0.2034 to 0.2412	0.1940 to 0.2375	0.1824 to 0.2272	0.1764 to 0.2295	0.2930 to 0.3332	0.2599 to 0.2945	0.2233 to 0.2518	0.2230 to 0.2563	0.2132 to 0.2440	0.2035 to 0.2323
Goodness of Fit																		
Degrees of Freedom	212	189	212	212	212	212	212	212	212	212	212	212	212	212	212	212	212	212
R squared	0.984	0.9889	0.9776	0.9837	0.9824	0.9822	0.9843	0.9841	0.981	0.9732	0.9686	0.956	0.9901	0.9899	0.9902	0.9869	0.9873	0.9881

Supplementary Table 15: List of primers which were obtained from Microsynth AG, Switzerland and plasmids used in this study.

Primer/plasmid name	Sequence (5'-3')
oAB507	AGACTCGAGGGTACCTTATTTGTACAGTTCATCCA
oAB707	CTAACTTACATTAATTGCGTTGCGCTCTAGATTATTTGTATAGTTCATCCATGCCATGTG
oAB708	GGGGACTGTTGGGCGCCATCTCCTTGCATGCACTAGTGTTCTTTCCTGCGTTATCCCCTG
oAB819	AGAGGATCCGAAGAATACGGGATAGAACTGAATGGTTTCAAAAGGCGAAGAAGACAAC
oAB734	GGTAGGTGGTCGCGCGGTTAACTTGCTTCCTGGCGGTTCTGGAGGT
oAB736	GTCTTTCGACTGAGCCTTTCGTTTTATTTGATGCCTCCTAGGTTA
oAB810	AGAAGGAGGTCATACCCGTTTTTTTGGAAGGAGGTAAATTAATTA
oAB744	GACTTTCTTAGCAACAATCCCGTAGATGTCCTGAACGGTTTCACTACCTCCAGAACCGCC
oAB589	TAACCTAGGAGGCATCAAATAAAACG
oAB446	ACCTCCAGAACCGCCAGGAAGCAAGTTAACCGCGCG
oAB448	GGCGGTTCTGGAGGTAGTGAAACCGTTCAGGACATCTACGG
oAB809	TAATTAATTTACCTCCTTCCAAAAAAACGGGTATG
pAB150	as described in Baumschlager et al., 2017
pAB50	as described in Baumschlager et al., 2017
pAB50-11k	GTCGTTTGGTATGGCTTCATTCAGCTCCGGTTCCCAACGATCAAGGCGAGTTACATGATCCCCCATGTTG<br>TGCAAAAAAGCGGTTAGCTCCTTCGGTCCTCCGATCGTTGTCAGAAGTAAGTTGGCCGCAGTGTTATCA<br>CTCATGGTTATGGCAGCACTGCATAATTCTCTTACTGTCATGCCATCCGTAAGATGCTTTTCTGTGACTG<br>GTGAGTACTCAACCAAGTCATTCTGAGAATAGTGTATGCGGCGACCGAGTTGCTCTTGCCCGGCGTCAA<br>TACGGGATAATACCGCGCCACATAGCAGAACTTTAAAAGTGCTCATCATTGGAAAACGTTCTTCGGGGC<br>GAAAACTCTCAAGGATCTTACCGCTGTTGAGATCCAGTTCGATGTAACCCACTCGTGCACCCAACTGAT<br>CTTCAGCATCTTTTACTTTCACCAGCGTTTCTGGGTGAGCAAAAACAGGAAGGCAAAATGCCGCAAAAA<br>AGGGAATAAGGGCGACACGGAAATGTTGAATACTCATACTCTTCCTTTTTCAATCATGATTGAAGCATT<br>TATCAGGGTTATTGTCTCATGAGCGGATACATATTTGAATGTATTTAGAAAAATAAACAAATAGGTCAT<br>GACCAAAATCCCTTAACGTGAGTTTTCGTTCCACTGAGCGTCAGACCCCGTAGAAAAGATCAAAGGATC<br>TTCTTGAGATCCTTTTTTTCTGCGCGTAATCTGCTGCTTGCAAACAAAAAAACCACCGCTACCAGCGGTG<br>GTTTGTTTGCCGGATCAAGAGCTACCAACTCTTTTTCCGAAGGTAACTGGCTTCAGCAGAGCGCAGATA<br>CCAAATACTGTCCTTCTAGTGTAGCCGTAGTTAGGCCACCACTTCAAGAACTCTGTAGCACCGCCTACA<br>TACCTCGCTCTGCTAATCCTGTTACCAGTGGCTGCTGCCAGTGGCGATAAGTCGTGTCTTACCGGGTTGG<br>ACTCAAGACGATAGTTACCGGATAAGGCGCAGCGGTCGGGCTGAACGGGGGGTTCGTGCACACAGCCC<br>AGCTTGGAGCGAACGACCTACACCGAACTGAGATACCTACAGCGTGAGCTATGAGAAAGCGCCACGCT<br>TCCCGAAGGGAGAAAGGCGGACAGGTATCCGGTAAGCGGCAGGGTCGGAACAGGAGAGCGCACGAGG<br>GAGCTTCCAGGGGGAAACGCCTGGTATCTTTATAGTCCTGTCGGGTTTCGCCACCTCTGACTTGAGCGTC<br>GATTTTTGTGATGCTCGTCAGGGGGGCGGAGCCTATGGAAAAACGCCAGCAACGCGGCCTTTTTACGGT<br>TCCTGGCCTTTTGCTGGCCTTTTGCTCACATGTTCTTTCCTGCGTTATCCCCTGATTCTGTGGATAACCGT<br>ATTACCGCCTTTGAGTGAGCTGATACCGCTCGCCGCAGCCGAACGACCGAGCGCAGCGAGTCAGTGAG<br>CGAGGAAGCGGAAGAGCGCCTGATGCGGTATTTTCTCCTTACACATCTGTGCGGTATTTCACACCGCAT<br>ATATGGTGCACTCTCAGTACAATCTGCTCTGATGCCGCATAGTTAAGCCAGTATACACTCCGCTATCGCT<br>ACGTGACTGGGTCATGGCTGCGCCCCGACACCCGCCAACACCCGCTGACGCGCCCTGACGGGCTTGTCT<br>GCTCCCGGCATCCGCTTACAGACAAGCTGTGACCGTCTCCGGGAGCTGCATGTGTCAGAGGTTTTCACC<br>GTCATCACCGAAACGCGCGAGGCAGCTGCGGTAAAGCTCATCAGCGTGGTCGTGAAGCGATTCACAGA<br>TGTCTGCCTGTTCATCCGCGTCCAGCTCGTTGAGTTTCTCCAGAAGCGTTAATGTCTGGCTTCTGATAAA<br>GCGGGCCATGTTAAGGGCGGTTTTTTCCTGTTTGGTCACTGATGCCTCCGTGTAAGGGGGATTTCTGTTC<br>ATGGGGGTAATGATACCGATGAAACGAGAGAGGATGCTCACGATACGGGTTACTGATGATGAACATGC<br>CCGGTTACTGGAACGTTGTGAGGGTAAACAACTGGCGGTATGGATGCGGCGGGACCAGAGAAAAATCA

	CTCAGGGTCAATGCCAGCGCTTCGTTAATACAGATGTAGGTGTTCCACAGGGTAGCCAGCAGCATCCTG<br>CGATGCAGATCCGGAACATAATGGTGCAGGGCGCTGACTTCCGCGTTTCCAGACTTTACGAAACACGGA<br>AACCGAAGACCATTCATATTGTTGCTCAGGTCGCAGACGTTTTGCAGCAGCAGTCGCTTCACGTTCGCT<br>CGCGTATCGGTGATTCATTCTGCTAACCAGTAAGGCAACCCCGCCAGCCTAGCCGGGTCCTCAACGACA<br>GGAGCACGATCATGCTAGTCATGCCCCGCGCCCACCGGAAGGAGCTGACTGGGTTGAAGGCTCTCAAG<br>GGCATCGGTCGAGATCCCGGTGCCTAATGAGTGAGCTAACTTACATTAATTGCGTTGCGCTCTAGATTA<br>TTTGTATAGTTCATCCATGCCATGTGTAATCCCAGCAGCTGTTACAAACTCAAGAAGGACCATGTGGTCT<br>CTCTTTTCGTTGGGATCTTTCGAAAGGGCAGATTGTGTGGACAGGTAATGGTTGTCTGGTAAAAGGACA<br>GGGCCATCGCCAATTGGAGTATTTTGTTGATAATGGTCTGCTAGTTGAACGCTTCCATCTTCAATGTTGT<br>GTCTAATTTTGAAGTTAACTTTGATTCCATTCTTTTGTTTGTCTGCCATGATGTATACATTGTGTGAGTTA<br>TAGTTGTATTCCAATTTGTGTCCAAGAATGTTTCCATCTTCTTTAAAATCAATACCTTTTAACTCGATTCT<br>ATTAACAAGGGTATCACCTTCAAACTTGACTTCAGCACGTGTCTTGTAGTTCCCGTCATCTTTGAAAAAT<br>ATAGTTCTTTCCTGTACATAACCTTCGGGCATGGCACTCTTGAAAAAGTCATGCTGTTTCATATGATCTG<br>GGTATCTCGCAAAGCATTGAACACCATAACCGAAAGTAGTGACAAGTGTTGGCCATGGAACAGGTAGT<br>TTTCCAGTAGTGCAAATAAATTTAAGGGTAAGTTTTCCGTATGTTGCATCACCTTCACCCTCTCCACTGA<br>CAGAAAATTTGTGCCCATTAACATCACCATCTAATTCAACAAGAATTGGGACAACTCCAGTGAAAAGTT<br>CTTCTCCTTTACGCATAGATCTTTTCCTTATTACGCCTGCTGGCGAAAGGGGGATGTGCTGCAAGGCGAT<br>TAAGTTGGGTAACGCCCGGGTTTTACCAGTCACGACGTTGTAAAACGACGGCCAGTGAATCGTGTCAGT<br>GGTGTATCATTATAGGGAGTTATTCCGGCCTGACAAGAGGAATCAGGGGATAACGCAGGAAAGAACAC<br>TAGTGCATGCAAGGAGATGGCGCCCAACAGTCCCCCGGCCACGGGGCCTGCCACCATACCCACGCCGA<br>AACAAGCGCTCATGAGCCCGAAGTGGCGAGCCCGATCTTCCCCATCGGTGATGTCGGCGATATAGGCG<br>CCAGCAACCGCACCTGTGGCGCCGGTGATGCCGGCCACGATGCGTCCGGCGTAGCCTAGGTAATACGA<br>CTCACTATAGGGAGAGGATCCGAAGAATACGGGATAGAACTGAATGGTTTCAAAAGGCGAAGAAGACA<br>ACATGGCGATTATCAAGGAATTTATGCGTTTCAAGGTCCACATGGAAGGCAGCGTCAATGGTCACGAAT<br>TTGAAATTGAAGGCGAAGGTGAAGGCCGTCCGTATGAAGGCACCCAGACGGCAAAACTGAAGGTCACC<br>AAAGGCGGTCCGCTGCCGTTTGCTTGGGATATTCTGTCACCGCAATTCATGTATGGTTCGAAAGCGTAC<br>GTTAAGCATCCGGCCGATATCCCGGACTATCTGAAACTGTCCTTTCCGGAAGGCTTCAAATGGGAACGT<br>GTTATGAACTTCGAAGATGGCGGTGTGGTTACCGTCACGCAGGATAGCTCTCTGCAAGACGGTGAATTT<br>ATTTATAAAGTGAAGCTGCGCGGCACCAATTTCCCGAGCGATGGTCCGGTTATGCAGAAAAAGACGAT<br>GGGCTGGGAAGCGAGTTCCGAACGTATGTACCCGGAAGACGGTGCCCTGAAAGGCGAAATCAAGCAGC<br>GCCTGAAACTGAAGGATGGCGGTCACTATGACGCAGAAGTGAAAACCACGTACAAGGCTAAAAAGCCG<br>GTCCAACTGCCGGGTGCATACAACGTGAACATCAAGCTGGATATCACCAGCCATAACGAAGACTATAC<br>GATCGTTGAACAGTACGAACGTGCAGAAGGCCGCCACTCTACCGGCGGTATGGATGAACTGTACAAAT<br>AAGGTACCCTCGAGTCTGGTAAAGAAACCGCTGCTGCGAAATTTGAACGCCAGCACATGGACTCGTCTA<br>CTAGTCGCAGCTTAATTAACCTAAACTGCTGCCACCGCTGAGCAATAACTAGCATAACCCCTTGGGGCC<br>TCTAAACGGGTCTTGAGGGGTTTTTTGCTAGCGAAAGGAGGAGTCGACTATATCCGGATTGGCGAATGG<br>GACGCGCCCTGTAGCGGCGCATTAAGCGCGGCGGGTGTGGTGGTTACGCGCAGCGTGACCGCTACACTT<br>GCCAGCGCCCTAGCGCCCGCTCCTTTCGCTTTCTTCCCTTCCTTTCTCGCCACGTTCGCCGGCTTTCCCCG<br>TCAAGCTCTAAATCGGGGGCTCCCTTTAGGGTTCCGATTTAGTGCTTTACGGCACCTCGACCCCAAAAA<br>ACTTGATTAGGGTGATGGTTCACGTAGTGGGCCATCGCCCTGATAGACGGTTTTTCGCCCTTTGACGTTG<br>GAGTCCACGTTCTTTAATAGTGGACTCTTGTTCCAAACTGGAACAACACTCAACCCTATCTCGGTCTATT<br>CTTTTGATTTATAAGGGATTTTGCCGATTTCGGCCTATTGGTTAAAAAATGAGCTGATTTAACAAAAATT<br>TAACGCGAATTTTAACAAAATATTAACGTTTACAATTTCTGGCGGCACGATGGCATGAGATTATCAAAA<br>AGGATCTTCACCTAGATCCTTTTAAATTAAAAATGAAGTTTTAAATCAATCTAAAGTATATATGAGTAA<br>ACTTGGTCTGACAGTTACCAATGCTTAATCAGTGAGGCACCTATCTCAGCGATCTGTCTATTTCGTTCAT<br>CCATAGTTGCCTGACTCCCCGTCGTGTAGATAACTACGATACGGGAGGGCTTACCATCTGGCCCCAGTG<br>CTGCAATGATACCGCGAGACCCACGCTCACCGGCTCCAGATTTATCAGCAATAAACCAGCCAGCCGGA<br>AGGGCCGAGCGCAGAAGTGGTCCTGCAACTTTATCCGCCTCCATCCAGTCTATTAATTGTTGCCGGGAA<br>GCTAGAGTAAGTAGTTCGCCAGTTAATAGTTTGCGCAACGTTGTTGCCATTGCTACAGGCATCGTGGTG<br>TCACGCTC

Supplementary References

1. Baumschlager, A., Aoki, S. K. & Khammash, M. Dynamic Blue Light-Inducible T7 RNA Polymerases (Opto-T7RNAPs) for Precise Spatiotemporal Gene Expression Control. *ACS Synth Biol* **6**, 2157–2167 (2017).
2. Vaidya, A. T., Chen, C.-H. H., Dunlap, J. C., Loros, J. J. & Crane, B. R. Structure of a light-activated LOV protein dimer that regulates transcription. *Sci Signal* **4**, 1–8 (2011).
