# Supplementary material for: Enhancing the performance of Magnets photosensors
Source: Nat Commun. 2026 Mar 18;17:4138. doi: 10.1038/s41467-026-70695-7 (PMC13149671; doi:10.1038/s41467-026-70695-7)
Supplement: Supplementary file 2 — Reporting Summary [file 41467_2026_70695_MOESM2_ESM.pdf]

nature portfolio

Corresponding author(s): Armin Baumschlager, Mustafa Khammash

Last updated by author(s): Feb 28, 2026

Reporting Summary

Nature Portfolio wishes to improve the reproducibility of the work that we publish. This form provides structure for consistency and transparency in reporting. For further information on Nature Portfolio policies, see our Editorial Policies and the Editorial Policy Checklist.

Statistics

For all statistical analyses, confirm that the following items are present in the figure legend, table legend, main text, or Methods section.

n/a	Confirmed	
[ ]	[x]	The exact sample size (*n*) for each experimental group/condition, given as a discrete number and unit of measurement
[ ]	[x]	A statement on whether measurements were taken from distinct samples or whether the same sample was measured repeatedly
[ ]	[x]	The statistical test(s) used AND whether they are one- or two-sided<br>*Only common tests should be described solely by name; describe more complex techniques in the Methods section.*
[x]	[ ]	A description of all covariates tested
[x]	[ ]	A description of any assumptions or corrections, such as tests of normality and adjustment for multiple comparisons
[ ]	[x]	A full description of the statistical parameters including central tendency (e.g. means) or other basic estimates (e.g. regression coefficient) AND variation (e.g. standard deviation) or associated estimates of uncertainty (e.g. confidence intervals)
[ ]	[x]	For null hypothesis testing, the test statistic (e.g. *F*, *t*, *r*) with confidence intervals, effect sizes, degrees of freedom and *P* value noted<br>*Give P values as exact values whenever suitable.*
[x]	[ ]	For Bayesian analysis, information on the choice of priors and Markov chain Monte Carlo settings
[x]	[ ]	For hierarchical and complex designs, identification of the appropriate level for tests and full reporting of outcomes
[x]	[ ]	Estimates of effect sizes (e.g. Cohen's *d*, Pearson's *r*), indicating how they were calculated

Our web collection on statistics for biologists contains articles on many of the points above.

Software and code

Policy information about availability of computer code

Data collection	CytExpert 2.1.092 (Beckman Coulter), Tecan Infinite 200Pro and Firmware v. 3.40
Data analysis	CytExpert 2.1.092 (Beckman Coulter), Microsoft Excel for Mac Version 16.16.9, PyMOL (Molecular Graphics System, Version 2.5.2 Schrödinger, LLC), R and packages as described in the methods section, Prism 9 for MacOS (Version 9.3.1 (#350, GraphPad Software, LLC.)

For manuscripts utilizing custom algorithms or software that are central to the research but not yet described in published literature, software must be made available to editors and reviewers. We strongly encourage code deposition in a community repository (e.g. GitHub). See the Nature Portfolio guidelines for submitting code & software for further information.

Data

Policy information about availability of data

All manuscripts must include a data availability statement. This statement should provide the following information, where applicable:

- Accession codes, unique identifiers, or web links for publicly available datasets
- A description of any restrictions on data availability
- For clinical datasets or third party data, please ensure that the statement adheres to our policy

Source data are provided with this paper and are available at Zenodo [https://doi.org/10.5281/zenodo.18815874]. PDB 3RH8 [https://doi.org/10.2210/pdb3RH8/pdb].

Research involving human participants, their data, or biological material

Policy information about studies with human participants or human data. See also policy information about sex, gender (identity/presentation), and sexual orientation and race, ethnicity and racism.

Reporting on sex and gender	Not applicable, no humans studied.
Reporting on race, ethnicity, or other socially relevant groupings	Not applicable, no humans studied.
Population characteristics	Not applicable, no humans studied.
Recruitment	Not applicable, no humans studied.
Ethics oversight	Not applicable, no humans studied.

Note that full information on the approval of the study protocol must also be provided in the manuscript.

Field-specific reporting

Please select the one below that is the best fit for your research. If you are not sure, read the appropriate sections before making your selection.

☒ Life sciences ☐ Behavioural & social sciences ☐ Ecological, evolutionary & environmental sciences

For a reference copy of the document with all sections, see nature.com/documents/nr-reporting-summary-flat.pdf

Life sciences study design

All studies must disclose on these points even when the disclosure is negative.

Sample size	Each experiment was performed at least three times and is on par with standard bacterial/mammalian cell experiments reported in the field. Sample size for variant screening was determined by the plasmid library sizes, which in turn were determined based on colony counts of dilution series to 1.2 * 106 and 1.9 * 106 for pMag and nMagHigh respectively. The mutation rate was estimated at 1.3-2.9 per kilobase pair through Sanger sequencing.<br>For variant characterization, sample sized were not predetermined, but the number of samples oriented at typical sample sizes in the field, meaning >= 3, which was the case for all characterizations and evaluated for statistical significance.
Data exclusions	Data exclusion was performed as described in 'Spectrophotometric and fluorometric measurements" to account for outliers.
Replication	A minimum of three biological replicates were used and are indicated in the figure legends. Further, all variants were analzed through spectrofluorometry and flow cytometry and in separate biological replicates, being in agreement. All findings were reliably reproducible.
Randomization	This is not relevant to our study, as one wild-type strain was compared to a number of mutants. All samples were treated exactly the same to determine the differences between the WT and the mutants in each tested condition (illumination, temperature, culture density).<br>Cell cultures were propagated from isogenic stocks derived from randomly selected colonies. When splitting cultures into different conditions as well as sampling cultures for measurements, cells within the cultures are randomly sampled as there is no control over which cells are selected.
Blinding	Researchers were not blinded during experiments because the experiments were not based on qualitative scoring metrics. In addition, each bacterial culture measurement is a random sampling of cells within a culture with no selection bias.

Reporting for specific materials, systems and methods

We require information from authors about some types of materials, experimental systems and methods used in many studies. Here, indicate whether each material, system or method listed is relevant to your study. If you are not sure if a list item applies to your research, read the appropriate section before selecting a response.

Materials & experimental systems

n/a	Involved in the study	
☒	☐	Antibodies
☐	☒	Eukaryotic cell lines
☒	☐	Palaeontology and archaeology
☒	☐	Animals and other organisms
☒	☐	Clinical data
☒	☐	Dual use research of concern
☒	☐	Plants

Methods

n/a	Involved in the study	
☒	☐	ChIP-seq
☐	☒	Flow cytometry
☒	☐	MRI-based neuroimaging

Eukaryotic cell lines

Policy information about cell lines and Sex and Gender in Research

Cell line source(s)	HEK293T cells (ATCC, strain number CRL-3216)
Authentication	The cell line used was not authenticated.
Mycoplasma contamination	Cells in culture were tested once for mycoplasma contamination with negative result.
Commonly misidentified lines (See ICLAC register)	In our study we only used HEK293T cells (strain number CRL-3216) received from ATCC. To our knowledge those were not contaminated cells.

Plants

Seed stocks	Not applicable, no seed stocks or plant material used.
Novel plant genotypes	Not applicable, no novel plant genotypes produced.
Authentication	Not applicable, no seed stocks or plant material used.

Flow Cytometry

Plots

Confirm that:

- [x] The axis labels state the marker and fluorochrome used (e.g. CD4-FITC).
- [x] The axis scales are clearly visible. Include numbers along axes only for bottom left plot of group (a 'group' is an analysis of identical markers).
- [x] All plots are contour plots with outliers or pseudocolor plots.
- [x] A numerical value for number of cells or percentage (with statistics) is provided.

Methodology

Sample preparation	Fluorescence measurements: Cells grown in minimal media were sampled and mixed with the same volume of 500 µg/ ml rifampicin and 50 µg/ ml tetracycline in phosphate-buffered saline. mCherry fluorescence was matured for 90 min at 37°C and read on the flow cytometer.
Instrument	Cytoflex S flow cytometer (Beckman Coulter) equipped with CytExpert 2.1.092 software.
Software	CytExpert 2.1.092 (Beckman Coulter)
Cell population abundance	Sorting was performed for selection of cells as part of the engineering strategy and described in the methods.
Gating strategy	mCherry fluorescence was measured with a 561 nm laser and 610/20 nm band pass filter and following gain settings: forward scatter 100, side scatter 100, mCherry gain 300. Thresholds of 2,500 FSC-H and 1,000 SSC-H were used for all samples. The flow cytometer was calibrated before each experiment with QC beads (CytoFLEX Daily QC Fluorospheres, Beckman Coulter) to ensure comparable fluorescence values across experiments from different days. Events were recorded in a two-dimensional forward and side scatter gate, which was drawn by eye and corresponded to the experimentally determined size of the testing strain at logarithmic growth and was kept constant for analysis of all experiments and used for calculations of the fluorescence mean using the CytExpert software.

- [x] Tick this box to confirm that a figure exemplifying the gating strategy is provided in the Supplementary Information.
